# Reduced vascular leakage correlates with breast carcinoma T regulatory cell infiltration but not with metastatic propensity

**DOI:** 10.1002/1878-0261.70144

**Published:** 2025-10-16

**Authors:** Liqun He, Chiara Testini, Neda Hekmati, Altea Bonello, Aglaia Schiza, Emmanuel Nwadozi, Mia Phillipson, Carina Strell, Michael Welsh

**Affiliations:** ^1^ Department of Immunology, Genetics and Pathology Uppsala University Sweden; ^2^ Department of Medical Cell Biology Uppsala University Sweden; ^3^ Department of Clinical Medicine University of Bergen Norway

**Keywords:** breast cancer, metastasis, *Shb*‐gene, T regulatory cells, vascular leakage, vascular normalization

## Abstract

The vasculature and the immune system both play roles in breast cancer progression and metastasis. In an experimental mouse model of *Shb*‐gene deficiency in endothelial cells, breast cancer lung metastasis correlated with immune suppression rather than with vascular leakage. The present study aimed to assess underlying gene expression changes in endothelial and immune cells responsible for this phenotype and to explore their relationship to human disease. Mouse endothelial cell *Shb*‐gene deficiency, leading to ‘vessel normalization’, resulted in altered expression of chemo/cytokine genes and upregulation of immune checkpoint genes in immune cells. Endothelial cells under these conditions exhibited gene expression patterns compatible with reduced angiogenesis and vascular leakage. Additionally, genes whose products relate to immune cell vascular transmigration and function were affected. In a human triple‐negative breast cancer cohort, tumors with reduced vascular leakage exhibited a higher relative proportion of regulatory T cells and larger tumor size. However, these changes were not associated with increased metastasis. In conclusion, a low leakage vascular phenotype reduces tumor cell intravasation/metastasis and modifies the immune response, which in the current context becomes pro‐tumoral.

## Graphical abstract

A mouse model for vascular normalization and a human breast cancer cohort were studied to understand the relationship between vascular leakage and tumor immune suppression. For this, endothelial and immune cell RNAseq, staining for vascular function, and immune cell profiling were employed. The data reveal that low leakage correlated with stronger tumor immune suppression.

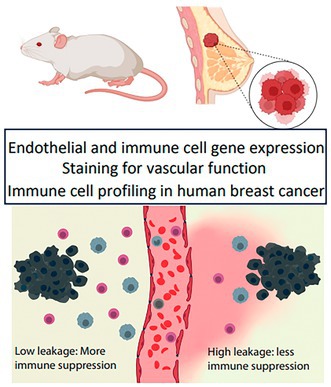

**Abbreviations**
CCR: chemokine receptor
CD: cluster of differentiation
CTLA4: cytotoxic T‐lymphocyte‐associated protein 4
DC: dendritic cells
DEG: differentially expressed genes
EC: endothelial cell
ECM: extracellular matrix
FACS: fluorescence‐activated cell sorting
GO: gene ontology
IC: immune cell
iECKO: conditional deletion in EC
IFN: interferon
KO: knockout
MDSC: myeloid‐derived suppressor cells
NK: natural killer
PD1: programmed cell death protein 1
pDC: plasmacytoid dendritic cell
PD‐L1: programmed death ligand 1
PD‐L2: programmed death ligand 2
SHB: Src‐homology domain protein B
TEM: transendothelial migration
TNBC: triple negative breast cancer
Treg: T regulatory cell
UMAP: uniform manifold approximation and projection
VEGFA: vascular endothelial growth factor A

## 1 Introduction

Cancer is a debilitating disease, with tumor metastasis being the most common cause of death. Tumor expansion and metastasis are complicated processes that are highly individual to each tumor, involving an interplay between the tumor cells and the stroma [1]. Tumor cells commonly evade novel antigen recognition and consecutive targeting by the adaptive immune system. This can be achieved by inducing reduced antigen presentation by antigen‐presenting cells [2], suppression of CD4^+^ T helper cells [3] or suppression of CD8^+^ T killer cells [4] due to immune‐suppressing cells such as T regulatory cells (Tregs) [5]. Additionally, innate immunity may be tamed in a pro‐tumoral manner by recruitment of myeloid‐derived suppressor cells (MDSC) [6] or tumor‐associated macrophages [7] that exhibit tissue‐restorative macrophage‐2 characteristics [2, 8]. Moreover, the tumor vasculature develops angiogenic features, primarily driven by the increased production of vascular endothelial growth factor‐A (VEGFA) [9], to supply oxygen and nutrients. However, as a result, the tumor vasculature is commonly dysfunctional and leaky [10, 11, 12], and these characteristics play a role in tumor cell intravasation, hematogenic dissemination, and metastasis [13]. The vasculature also is a portal for immune cell extravasation to infiltrate the tumor, and for this reason, it can be expected that the vasculature plays a role in regulating the tumor's immune response [14].

There have been astounding recent developments in cancer treatment, including treatment with inhibitors of amplified tumoral signaling pathways, antiangiogenic treatment, and inhibition of immune checkpoint proteins [15]. Most successful has been immune checkpoint inhibition targeting the *CTLA4* gene product, PD‐L1 (*CD274*), and PD1 (*PDCD1*), resulting in the cure of a number of cancers [16]. Despite this success, many cancers remain refractory to immune checkpoint inhibition, and thus the treatment repertoire needs to be expanded [17]. Further candidates under investigation are PD‐L2 (*PDCD1LG2*), *ICOS*, *TIM3*, *TREM2*, *CD276*, *VISTA*, and *TIGIT* [18]. Concerning angiogenesis inhibition, most treatments only have transient, if any, effects at all [19]. This can be partially explained by ‘vascular normalization’ [12], the process in which the vasculature becomes less leaky with a lower vascular density but with increased functionality, thus not changing tumor oxygenation, or by resistance to angiogenic inhibition. Vascular normalization is a rapid process upon efficient antiangiogenic treatment and is apparent within one week [20].

In addition to supplying oxygen and nutrients, the vasculature also governs the tumor's infiltration of leukocytes by immune cell extravasation, which is a complex process requiring endothelial cell (EC)–immune cell (IC) cell surface receptor interactions, local chemokine/cytokine production, and regulation of EC adherens and tight junctions [21, 22]. It is thus clear that ECs play an important role in this context, although there is currently no simple understanding of possible mechanisms by which ECs may operate as ‘gate‐keepers’ to selectively permit or restrict infiltration of specific ICs and thus exert control of tumor immunity [14]. However, EC junctions play a role in this context, and VEGFA is a major regulator of adherens junction disassembly [11], which is followed by vascular leakage.

Tregs are important players with respect to anticancer immunity. Although their extravasation and tumor infiltration follow the general mechanisms of leukocyte transendothelial migration (TEM) as briefly described above, a certain degree of specificity with respect to leukocyte extravasation has been observed [14] although it is unclear to what extent such described specificity applies to Treg TEM. However, the local cytokine/chemokine environment is also important for Treg recruitment [23, 24], as is the local expression of immune checkpoint proteins [25]. Particularly, stimulation of the *Ccr5* chemokine receptor gene product appears important for Treg homing [26].

The SHB (Src homology 2 domain protein B) adapter protein is an important regulator of VEGFA‐induced angiogenesis and vascular leakage [27, 28]. Our recent study demonstrated that E0771.lmb breast carcinomas grown in mice with *Shb* conditionally deleted in ECs (*Shb* iECKO) exhibited reduced tumor vascular leakage while lung metastasis was simultaneously increased [29]. This paradox was attributed to selective infiltration of immune suppressive ICs into tumors as a consequence of the *Shb*‐deficient EC phenotype, demonstrating that ECs indeed exert selective control of the tumor's immune response.

Breast cancer exhibits the unusual trait of tumor recurrence at late time points after successful removal of the primary tumor. Additionally, the response of breast cancer to immune checkpoint protein blockade and angiogenesis inhibition is generally limited, although triple negative breast cancer may, under certain conditions, benefit from complementary immunotherapy treatment [30].

The present study was conducted in order to obtain a molecular explanation for the underlying mechanism(s) governing selective infiltration of immune‐suppressive ICs as a consequence of vascular normalization, with particular focus on breast cancer. For this purpose, mouse tumor ICs and ECs from *Shb* iECKO mice were subjected to RNAseq analysis. In addition, surgical specimens of human triple negative breast cancer were stained for IC, EC, and leakage markers. The data suggest reduced vascular leakage, without concomitant alterations of hypoxia, as an explanation for increased tumor immune suppression resulting from altered EC function. Metastasis in human disease, however, appears predominantly dependent on increased vascular leakage.

## 2 Materials and methods

### 2.1 Mouse experiments

Animal experimentation was approved by the animal ethics committee at Uppsala County Court (5.8.18‐05732_2022). Up to five mice were housed in cages of 501 cm^2^ at 24 °C at 40–60% humidity with free access to water and standard chow at the animal department at the Biomedical Centre at Uppsala University. Female mice at 10–16 weeks of age were used for the experiments. E0771.lmb tumors (RRID:CVCL_BOA2) were grown orthotopically in the forth mammary fat pad after tamoxifen injection (to induce *Shb* floxing) as previously described in wild‐type and *Shb* iECKO (*Shb* flox/flox; *Cdh5*‐CreERt2) mice [29], alternatively in *Shb* ICKO (*Shb* flox/flox; *Vav1*‐cre) mice. The conditional Shbflox (Shbtm1a(EUCOMM)Hmgu) mouse on the C57Bl/6 strain was generated by the Intrafrontier I3 mouse project at the Biocenter Transgenic Core Facility, University of Oulu, Oulu, Finland. The Cdh5‐CreERT2 transgenic mouse was kindly provided by Dr Ralf Adams, Max Planck Institute for Molecular Biomedicine, Münster, Germany. The Vav1‐cre mouse was kindly provided by Dr Maria Ulvmar, Uppsala University.

Mice were sacrificed on day 14–16 after tumor cell injection. The tumors were digested, CD45^+^‐cells isolated by MACS™ microbeads (Miltenyi, Bergich Gladbach, Germany) [29] and subsequently CD31^+^‐cells isolated by fluorescence‐activated cell sorting (FACS) [31] after staining for CD31. The average wild‐type tumor weight was 0.13 ± 0.03 mg and the corresponding *Shb* iECKO value was 0.11 ± 0.03 mg at sacrifice. The average number of CD45^+^ cells isolated from the wild‐type tumors was 2.4 ± 0.4 × 10^6^ whereas 2.6 ± 0.8 × 10^6^ cells were isolated from the *Shb* iECKO tumors.

### 2.2 RNAseq

Sequencing libraries were prepared from 10 000 cells after recovery using the Chromium Next GEM Single Cell 3′ Reagent kit v3.1 with dual index (cat# 1000268/269/120/127/1000215, 10× Genomics) according to the manufacturer's protocol (User guide CG000315, Chromium Next GEM Single Cell 3′ Reagent Kits v3.1 (Dual Index), 10× Genomics). The libraries were sequenced with the NovaSeq 6000 system (Paired‐end 150 bp read length, S4 flowcell, and v1.5 sequencing chemistry).

The raw sequence data were processed using cell ranger software (version: 4.0.0; 10x Genomics Inc., Pleasanton, CA, USA). The reads were aligned to mouse genome mm10 and gene counts matrix was summarized for each sample. The raw gene counts data were then loaded into r seurat package (version: 4.3.0; Satija Lab, New York Genome Center, New York, NY, USA) for quality control and downstream analysis and visualization. The cells which have less than 500 detected genes or have mitochondrial genes greater than 10% were filtered out. For the remaining cells, a scale factor of 10 000 was used to normalize the raw gene expression counts. To correct for the batch effect among different samples, the CCA method with default settings in the seurat package was applied to integrate all the samples. The top 2000 variable genes in the data were obtained using the vst method and the first 30 principal components were used for the shared nearest neighbor clustering analysis and Uniform Manifold Approximation and Projection (UMAP) visualization. For clustering the cells, the FindClusters function was used (with resolution *r* = 1). The genes enriched in each cluster were then subsequently identified using FindAllMarkers function. It first applies a Wilcoxon Rank Sum test for differential expression and then performs multiple test correction using Bonferroni correction method. The multiple test corrected *P* value < 0.05 was used as cutoff to identify significant differentially expressed genes.

Since the cluster cell numbers differed between the different experiments, only effects that were consistently observed in the three experiments were included. Furthermore, since the Cdh5‐creERt2 transgene used for conditionally deleting *Shb* in EC may also conditionally delete *Shb* in certain populations of ICs [31], effects on gene expression observed with Vav1‐cre floxing of *Shb* (*Shb* ICKO) were also excluded.

To visualize markers in different clusters, the DotPlot function in seurat package and also pheatmap package (version: 1.0.12) were applied. To show the detailed gene expression difference between groups, the r ggplot2 package (version: 3.5.1) was used to generate the volcano plot. To illustrate the interactions between different cell types, the r ccplotr package (version: 0.99.3) was utilized to produce the circular cell–cell interaction networks by employing literature‐assembled ligand–receptor pairs [32]. The *Cdh5‐creERt2*/*Shb* ^*flox/flox*^ data (iECKO) can be found at https://heomics.shinyapps.io/MW_Cdh5‐creERt2_Shbflox and the *Vav‐cre*/*Shb* ^flox/flox^ data at https://heomics.shinyapps.io/MW_Vav‐cre_Shbflox/.

Total (bulk) EC RNA was isolated using the RNeasy mini‐kit (Qiagen, Hilden, Germany) from CD31^+^ cells previously depleted of CD45^+^ cells and subjected to bulk RNAseq using NovaSeq6000 or NovaSeq X Plus Sequencing. Libraries were prepared from 30 ng total RNA using the Illumina Stranded Total RNA library preparation kit with Ribo‐Zero Plus treatment (cat# 20040525/20040529, Illumina Inc., San Diego, CA, USA). Unique dual indexes (cat# 20040553/20040554, Illumina Inc.) were used. The library preparation was performed according to the manufacturer's protocol (#1000000124514). Paired‐end 150 bp read length were sequenced. The RNAseq data were analyzed with the nf‐core/rnaseq bioinformatics pipeline. Sequencing was done in 4 separate experiments with 2–5 samples per genotype in each experiment including samples that were resequenced (total 11 WT samples from 9 tumors and 10 KO samples from 8 tumors). Average expression for each gene per experiment and genotype were calculated and compared by paired Student's *t*‐test.

Sequencing was performed by the SNP&SEQ Technology Platform in Uppsala. The facility is part of the National Genomics Infrastructure (NGI) Sweden and Science for Life Laboratory. The SNP&SEQ Platform is also supported by the Swedish Research Council and the Knut and Alice Wallenberg Foundation.

### 2.3 Human breast cancer cohort

A cohort of human triple negative breast cancer (TNBC) patients diagnosed between 2014 and 2022 at Uppsala Akademiska University Hospital was employed to study the expression of IC and vascular markers in 20 tumors. The study was conducted in accordance with the Declaration of Helsinki. Breast tumor tissue samples were identified, collected, and analyzed as approved by the Swedish Ethical Review Authority (permit number Dnr2022‐00478‐01 with amendment Dnr2022‐06689‐02 including informed consent for each patient via U‐CAN/Dnr2010/198/3). We selected TNBC due to its complex metastasis and immune evasion mechanisms, making it an ideal model to study the role of vascular leakage and immune cell infiltration in tumor progression. Additionally, TNBC is one of the few subtypes that may respond to immunotherapy and VEGF inhibitors, providing an opportunity to explore how these factors influence treatment outcomes. We selected 10 cases with metastatic recurrence and 10 without recurrence to facilitate a more balanced comparison of tumor behavior. The tumors were selected in a manner to minimize differences between the groups with respect to age, tumor size, and lymph node status. The treatment schemes differ between patients with or without lymph node involvement and metastasis, and therefore these are potentially confounding factors. This we had no means of influencing since the treatment regimens were based on the medical history. This approach enables us to explore how factors like vascular leakage and immune cell infiltration differ between patients who experience relapse and those who remain disease‐free, offering valuable insights into the mechanisms driving disease progression and recurrence.

### 2.4 Multiplex immunofluorescence

The multiplex staining was performed with the BOND RX autostainer (Leica Biosystems). Paraffin‐embedded sections (4 μm) were deparaffinized with Dewax solution (Leica Bond #AR9222, Leica Biosystems, Nussloch, Germany) followed by first antigen retrieval using Epitope retrieval solution 2 (Leica Bond ER2, #AR9640) at the final temperature of 95 °C and for a total of 30 min. Staining started by performing six subsequent staining cycles following the antibody orders for panel 1 and 2 respectively: VE‐cadherin, PD‐L1, Fibrinopeptide A, CD4, FoxP3, CTLA4 and PD‐L1, PD‐1, Granzyme B, CD163, CD20, CD8. Details of antibodies are provided in Table S8. Each staining cycle was followed by 5 min blocking with Protein Block Serum‐Free ready to use (DAKO #X0909). Primary antibody was added and incubated for 30 min followed by 10 min incubation with secondary antibody either ImmPress‐mouse HRP, MP‐7402, or ImmPress‐rabbit HRP, MP‐7401 (Vector Laboratories, Newark, CA, USA). At the end of a 10‐min incubation with tyramide signal amplification by using opal dyes in the following order for panel 1 and 2 respectively: 690, 570, 520, 480, 620, TSA‐DIG/780 and 690, 620, 520, 570, 480 and TSA‐DIG/780 (all Akoya Biosciences). All opal dyes were diluted at 1 : 300 in 1× Automation Amplification Diluent (Akoya Biosciences #FP1609, Marlborough, MA, USA). Each cycle was completed with antigen retrieval by using Epitope retrieval solution 1 (Leica Bond ER1, #AR9961) for 20 min at 95 °C. All slides were counterstained with DAPI for nuclear staining for 5 min (Akoya Biosciences, #FP1490) and followed by mounting with ProLong Diamond Antifade media (Thermo Fisher Scientific, #P36970, Waltham, MA, USA). All staining steps were performed at room temperature and Wash solution (Leica Bond, #AR9590) was used for washing steps.

Stained sections were scanned and spectrally unmixed by using Vectrapolaris© system and phenoimager‐ht‐2 software. Cell detection was performed on Qupath with a set intensity threshold of 100, a background radius of 8 μm, a sigma factor of 1.5, and cell expansion of 5 μm. Based on visual inspection, these settings accurately detected cells consistently across all images. Cell numbers were determined per tumor unit area in arbitrary units. Fibrinogen peptide A (FpA) staining was determined as a positive percentage area per tumor area.

### 2.5 Statistics

prism 10 was used for statistical comparisons between groups of data using Student's *t*‐test (paired or unpaired whenever appropriate) or correlation analysis. Paired *t*‐tests were considered appropriate when conditions of the two experimental groups in a separate experiment were identical, that is, same tumor and RNAseq experiment. Correction was done for multiple comparisons for scRNAseq data except for selected gene expression (15 genes) in which TPM per cluster (as indicated) and sample were compared between wild type and knockout. Bulk RNAseq and correlation analyses were not corrected for multiple comparisons. The uncorrected data were used to prioritize gene expression pattern changes or possible correlation patterns.

## 3 Results

### 3.1 IC single‐cell RNAseq (scRNAseq)

**Fig. 1:**
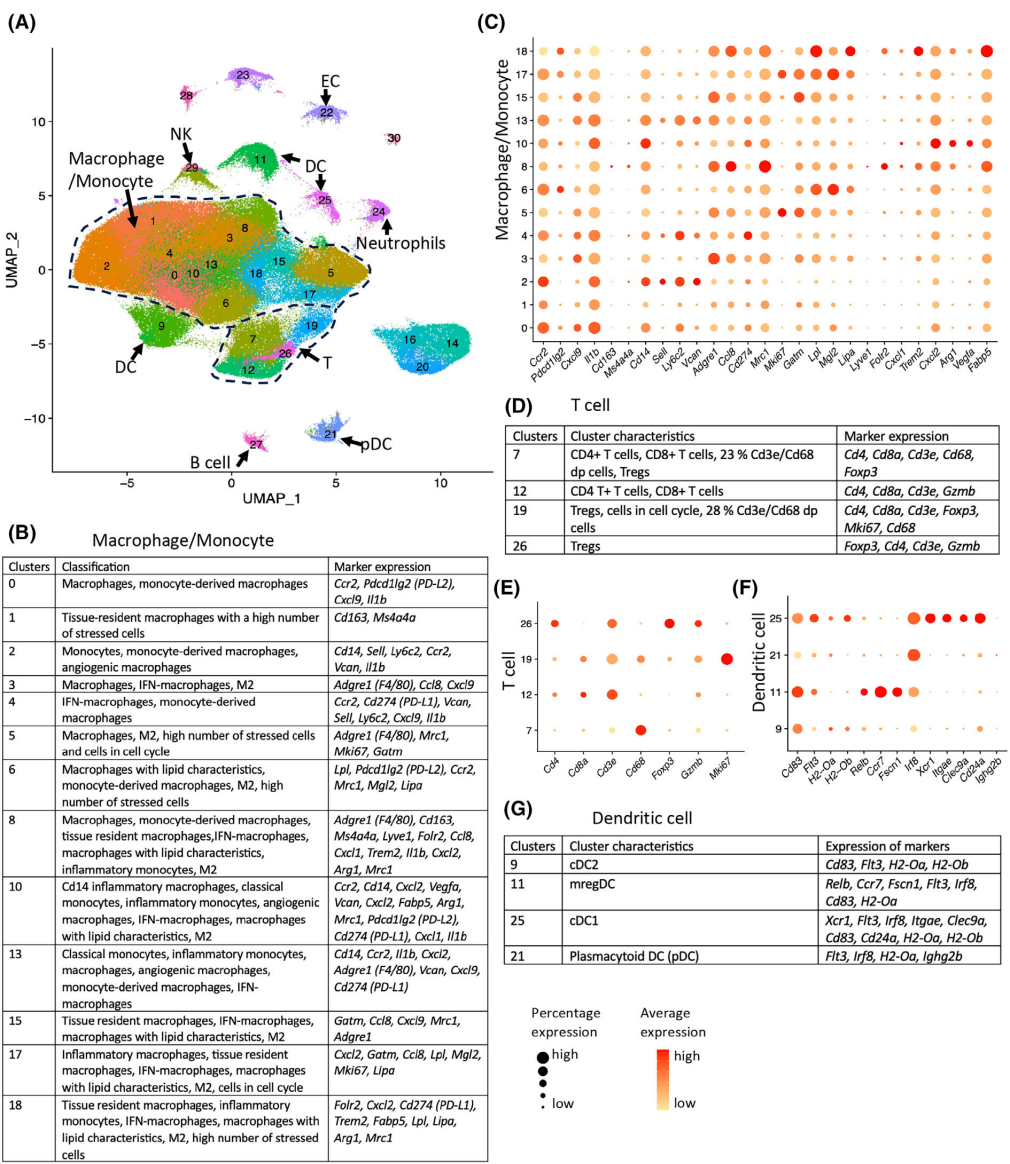
Breast cancer IC cluster characterization by markers. (A) UMAP1/UMAP2 plot depicting cluster separation. Clusters 14, 16, 20, and 23 are probably fibroblasts, 28 mural cells, and 30 epithelial cells. (B) Macrophage/monocyte clusters, their classification, and markers motivating the classification. (C) Relative expression of myeloid markers in the macrophage/monocyte clusters. (D) T cell clusters, cluster characteristics, and their markers. (E) Relative expression of the T cell markers. (F) Relative expression of the dendritic (DC) markers. (G) DC clusters, cluster characteristics, and markers. EC, endothelial cells; IC, immune cells; iECKO, conditional deletion of *Shb* in EC; IFN, interferon; M2, macrophage type 2; NK, natural killer cells; Treg, T regulatory cells; UMAP, uniform manifold approximation and projection. *N* = 6 each genotype.

To evaluate the specifics of the immune‐suppressive phenotype in the *Shb* iECKO E0771.lmb experimental breast cancer model, in which previously increased lung metastasis accompanied by increased tumor immune suppression and reduced leakage was demonstrated [29], scRNAseq on CD45^+^ cells was conducted in three separate experiments with tumor samples from two wild‐type and two *Shb* iECKO mice in each experiment (total of six separate tumor samples for each genotype). A total of 238 188 cells were sequenced (119 530 wild‐type cells and 118 658 *Shb* iECKO cells). seurat clustering identified 30 separate clusters. Clusters 0–6, 8, 10, 13, 15, 17, and 18 consisted of primarily macrophage/monocyte populations (163 864 cells combined), clusters 7, 12, 19, and 26 consisting of T cells (25 374 cells combined), 9, 11, 25 dendritic cells (DCs) (17 827 cells combined) [3, 33], 21 of plasmacytoid dendritic cells (pDC) (3188 cells) [33], 24 of neutrophils (2353 cells), 27 of B cells (1504 cells), and 29 of natural killer (NK) cells (696 cells) [3] (Fig. 1A for Umap1 vs. Umap2 depiction, Table S1 for cell number percentages). Clusters 14, 16, 20, and 23 appeared primarily to express fibroblast markers, 22 endothelial markers, 28 mural cell markers, and cluster 30 epithelial markers, and consequently, these were not analyzed further unlike the macrophage/monocyte, T cell, and DC clusters that were subjected to more detailed analysis.

Characterization of the macrophage/monocyte clusters (Fig. 1B,C) was inspired by a recent publication [34], describing tumor macrophage/monocyte cell characteristics according to specific markers defined in that study and in addition employing the macrophage 2 markers *Arg1*, *Mrc1*, and *Mgl2* (Fig. S1, Table S2). Expression of the macrophage 2 genes *Arg1*, *Mrc1*, and *Mgl2* was noted in clusters 3, 5, 6, 8, 10, 15, 17, and 18, although no distinct macrophage 2 profiles correlated with the *Shb* iECKO genotype. In addition, clusters with cells active in the cell cycle (*Mki67*) and/or stressed cells (clusters with more than 10 percent of the cells expressing more than 7.5% transcripts from mitochondrial DNA) were also taken into account. Clusters 5 and 17 contained cells active in the cell cycle, and clusters 1, 5, 6, and 18 contained a large proportion of stressed cells. It is apparent that the macrophage/monocyte clusters were pleiotropic with respect to the selected markers. Whereas clusters 2 and 13 seem to be the clusters mostly enriched for monocytes, these also expressed macrophage characteristics. The relative cell numbers were increased for cluster 4 and decreased for clusters 17 and 25 in response to the *Shb* iECKO genotype (Table S1). Clusters 8 and 10 contained macrophages with IFN (interferon) and lipid metabolism properties. The presence of macrophages with ‘IFN‐properties’ is a common feature in tumors, and thus, these cells are likely to have some tumor supportive features. Lipid macrophages are associated with poor prognosis, making these also populations of interest [34]. Cluster 4 showed an IFN profile and was the cluster with the highest expression of the immune checkpoint protein PD‐L1 gene (*Cd274*). This is relevant since the cell numbers of this cluster were increased as a consequence of the knockout genotype. Other clusters displaying lipid or IFN profiles were 3, 6, 13, 15, 17, and 18.

**Fig. 2:**
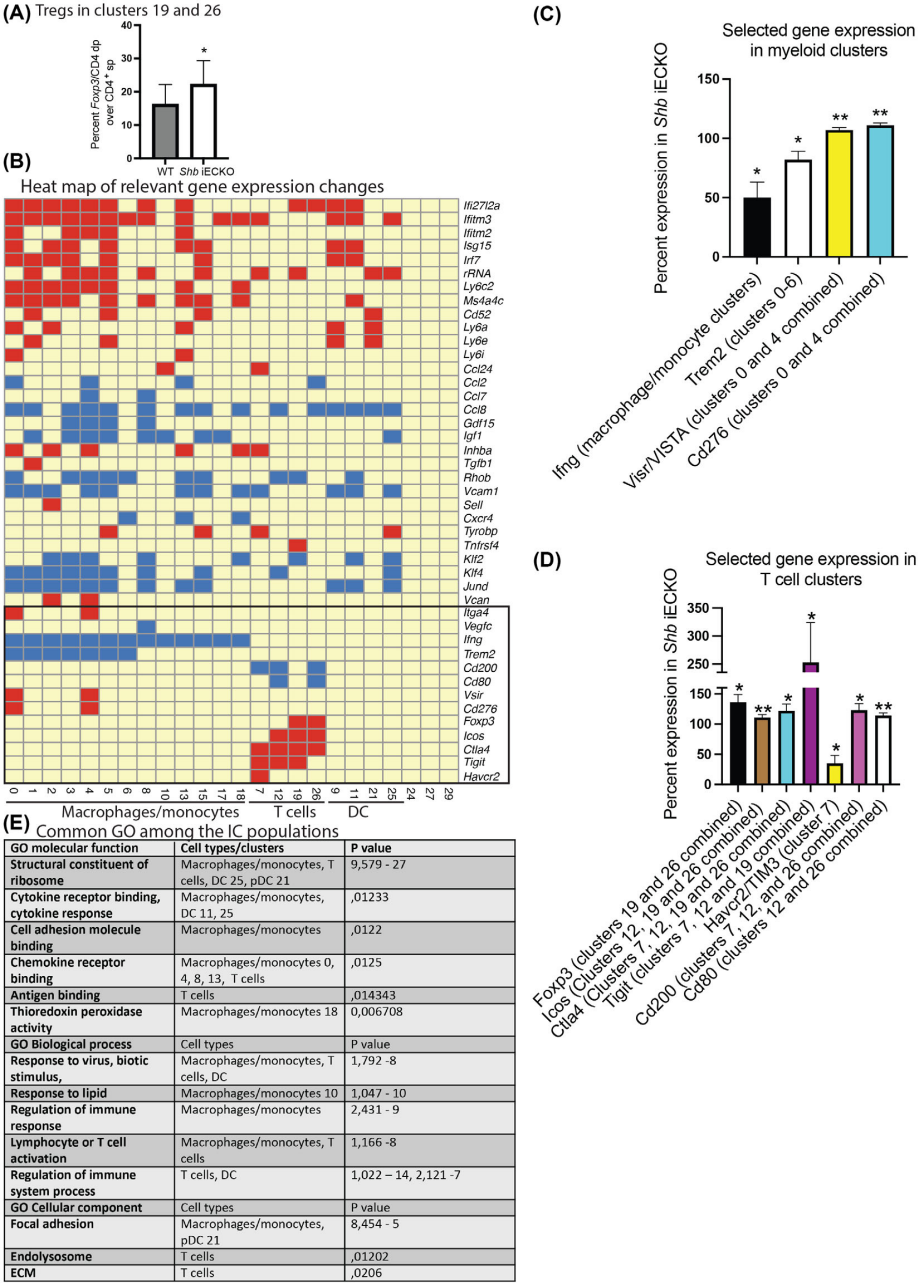
Differentially expressed genes (DEGs) in breast IC upon *Shb* iECKO. (A) Percentage Foxp3/Cd4 double‐positive cells among CD4^+^ cells in clusters 19 and 26. *Indicates *P* < 0.05 by pairwise comparison using a paired Students' *t*‐test in the three separate experiments based on six samples each genotype. Means ± SEM are given. (B) Heat map of certain significant gene expression changes in different clusters by a Wilcoxon Rank Sum test for differential expression and then multiple test correction using the Bonferroni correction method. The multiple test corrected *P* value < 0.05 was used as cutoff to identify significant differentially expressed genes. Boxed area displays genes with significant changes calculated on average expression in one or multiple clusters combined. Red, increased expression and blue, decreased expression. (C) *Shb* iECKO expression (percent of wild‐type control) of selected genes in myeloid clusters. * and ** indicate *P* < 0.05 and *P* < 0.01, respectively, when TPM data were pairwise compared by a paired Students' *t*‐test based on six samples for each genotype performed at three separate occasions. Means ± SEM are given. (D) *Shb* iECKO expression (percent of wild‐type control) of selected genes in T cell clusters. * and ** indicate *P* < 0.05 and *P* < 0.01, respectively, when TPM data were pairwise compared by a paired Students' *t*‐test based on six samples for each genotype performed at three separate occasions. Means ± SEM are given. (E) Common significant GO categories for the IC populations. DC, dendritic cells; ECM, extracellular matrix; GO, gene ontology; IC, immune cells; iECKO, conditional deletion of *Shb* in EC; Treg, T regulatory cells. *N* = 6.

*Cd4* was expressed in all four T cell clusters (7, 12, 19, and 26) whereas *Cd8a* was relatively sparsely expressed in cluster 26 (Fig. 1D,E and Fig. S2). Of these, cluster 19 contained many cells active in the cell cycle. Clusters 7 and 19 were rich in *Cd3e/Cd68* double‐positive (dp) cells (23% cluster 7 and 28% cluster 19, Fig. S2) although the average *Cd68* expression in these clusters was lower than in most macrophage clusters (Fig. S1). Upon activation, CD4^+^ and CD8^+^ T cells have been described to express macrophage markers [35, 36]. Clusters 19 and 26 contained Tregs by virtue of their expression of *Foxp3*. The percentages of *Foxp3* ^+^/*Cd4* ^+^ double‐positive cells of total *Cd4* ^+^ cells were 16 ± 6 in the wild‐type populations and 22 ± 7 in the *Shb* iECKO populations (*P* < 0.05 paired Student's *t*‐test for the three separate tumor experiments, Fig. 2A) suggesting immune‐suppressive conditions as a consequence of endothelial *Shb* deficiency.

DC cluster characterization is shown in Fig. 1F,G and Fig. S2. Cluster 25 contained primarily cDC1 cells, which comprise antigen‐presenting cells. The cell numbers of this cluster were significantly decreased in *Shb* iECKO mice, supporting the view of a reduced immune activation under these conditions [37]. Cluster 9 was primarily composed of cDC2 cells, and cluster 11 of mregDC cells. Cluster 21 consisted of pDC cells.

### 3.2 Differentially expressed IC genes (DEGs) as a consequence EC *Shb* deficiency

DEGs between wild‐type and *Shb* iECKO ICs were determined on the expression of genes in individual cells in each cluster. Table S3 shows all DEGs by cluster and for combined macrophage/monocyte, T cell, and DC clusters.

Selected significant gene expression changes have been summarized in Fig. 2B. A set of genes described as IFN‐induced (top five genes in Fig. 2B) can be detected as DEGs in most clusters, despite the fact that there was no increase in IFN expression among the cell populations. These genes have been described, according to Genbank (https://www.ncbi.nlm.nih.gov/gene/), to regulate virus entry, DNA binding, apoptosis, mitochondrial localization, and ubiquitin ligase. The consequences of their differential expression in immune cells for tumor biology remain unclear, although high levels of *Ifitm2* were observed in terminally exhausted T cells [38]. Increased expression of genes coding for ribosomal proteins was commonly observed, suggesting increased protein synthesis in tumor ICs from *Shb* iECKO mice [39]. Expression of *Ly6c2*, *Ms4a4c*, *Cd52*, *Ly6a*, *Ly6e*, and *Ly6i*, all with poorly defined function in the current context (https://www.ncbi.nlm.nih.gov/gene/), was increased in several clusters. Among other gene expression changes, *Inhba* was increased in several clusters. *Inhba* codes for activin A, which is an immune suppressive cytokine [5, 40]. Another immunosuppressive cytokine gene, *Tgfb1* [41], showed increased expression in cluster 1. We observed reduced *Igf1* expression in a number of myeloid clusters, and lower *Igf1* has been associated with the progression of breast cancer lung metastasis [42].

A set of chemokines (*Ccl2*, *Ccl4*, *Ccl5*, *Ccl7*, *Ccl8*, *Ccl12*, *Ccl22*, *Ccl24*, and *Cxcl10*) was conspicuously detected among the DEGs (Table S3). These interact in a largely overlapping manner with the chemokine receptors *Ccr1*, *Ccr2*, *Ccr3*, *Ccr4*, *Ccr5*, and *Cxcr3* [43, 44, 45] expressed in monocytes, macrophages, DCs, and T cells. Chemokines alter immune responses by recruiting various immune cells, and their effects are diverse depending on context [5, 46]. Consequently, the net effect of these changes on the immune response is difficult to predict [2]. Assessing chemokine receptor stimulation in an integrative manner based on individual chemokine expression, Ccr1 appears to receive both stimulatory and inhibitory cues, Ccr2 inhibitory cues, Ccr3 stimulatory and inhibitory cues, Ccr4 inhibitory cues (see below), and Ccr5 both stimulatory and inhibitory cues. In the cases where mixed signals are provided, the spatial relationship between the different cell types may be important. Ccr2, which appeared to be less active in the *Shb* knockout environment, is expressed on macrophages, T cells, and DCs, and thus the outcome of reduced stimulation is hard to predict. Whereas most chemokines were downregulated in several clusters, *Ccl5* expression was increased in the macrophage cluster 5, *Ccl24* in the T cell and macrophage clusters 7 and 10, and *Ccl22* in the mregDC‐containing cluster 11. *Ccl22* interacts selectively with *Ccr4* on T cells, including Tregs, and *Ccl22* expression in mregDC cells has recently been shown to recruit Tregs that prevent tumor antigen trafficking to the regional lymph nodes [47]. Thus, increased *Ccl22* expression in cluster 11 contributes to explaining the increased presence of Tregs, the reduction of local lymph node Tregs [29] and the reduction of the antigen‐presenting cluster 25 cDC1 population as a consequence of *Shb* iECKO. Operating in synergy with *Ccl22* in the current context is the increase of *Cxcl10*, a chemokine that participates in T cell recruitment [48], which was higher in the monocyte cluster 2. The *Cxcl10* cognate receptor is *Cxcr3* [49] and *Cxcr3*‐expressing Tregs interact with type cDC1 cells to restrict antitumor immunity [50] thus also contributing to explaining the reduction of cluster 25 cell numbers. Expression of *Ccl4* was reduced in the T cell cluster 19. Expression of *Ccl2* (macrophage/monocyte and Treg clusters), *Ccl7* (macrophage clusters), and *Ccl8* (macrophage/monocyte, T cell, and DC clusters) was reduced, and the latter showed the most prominent changes. *Ccl8* has been poorly studied, although it partakes in recruiting most categories of T cells, which may lead to an immunostimulatory effect [51], and thus, the reduction of *Ccl8* expression may promote an immune suppressive environment. Taken as a whole, the predominant effect is reduced chemokine expression, and this is compatible with a less inflammatory environment.

*Klf2*, *Kl4* (transcription factors), and *Gdf15* (cytokine) have been shown to resist inflammatory conditions [52, 53] whereas *Jund* codes for a transcription factor that is required for the production of inflammatory cytokines such as *Il1b* [54]. The reduced expression of these genes thus also suggests a less inflammatory state in the *Shb* iECKO tumors.

*Vcam1* codes for a cell adhesion molecule that interacts with the integrin *Itga4* and endothelial *Vcam1* partakes in leukocyte recruitment [14]. Macrophage *Vcam1* is presumably involved in IC/IC interactions, and *Vcam1*‐expressing macrophages are commonly present in tumors [7, 55]. In addition, *Vcam1*‐expressing macrophages also express the immune stimulatory product *Trem2* [55]. Thus, the reduced *Vcam1* expression is paralleled by decreased expression of *Trem2* in *Shb* iECKO macrophages (see below). *Vcan* codes for an extracellular matrix protein [56] that is expressed in angiogenic and monocyte‐derived macrophages [34] and is thus pro‐tumoral. *RhoB* was decreased in several clusters, and *RhoB* serves a role in endocytosis and antigen presentation [57].

Gene expression changes in the T cell and DC clusters that were not observed in the macrophage/monocyte clusters have also been listed in Table S3. *Il2ra* (CD25) is particularly relevant since elevated expression (37% in cluster 19) is compatible with more Tregs. *Ccl22* was among the DC‐specific changes, as discussed above.

Gene expression differences were also calculated for 15 selected genes of major importance to immune suppression based on average gene expression in each individual sample and cluster followed by paired comparisons between the corresponding wild‐type and *Shb* iECKO populations (Fig. 2C,D). According to these comparisons, 11 were significantly (individual *P* values < 0.05) different in relevant macrophage or T cell clusters. In macrophage/monocyte cells, the immune stimulatory gene products *Ifng* (50 ± 13% decrease in all macrophage/monocyte clusters when combined) [58] and *Trem2* (18 ± 7% decrease in combined clusters 0–6) [59] exhibited significantly reduced expression whereas the immune suppressive genes *Visr/VISTA* (7 ± 2% increase in combined 0 and 4 clusters) [60] and *Cd276* (11 ± 2% increase in combined 0 and 4 clusters) [61, 62] were significantly increased (Fig. 2C). In T cells, the Treg marker *Foxp3* (36 ± 16% increase in combined clusters 19 and 26) or the immune suppressing/immune checkpoint genes *Icos* (11 ± 5% increase in combined clusters 12, 19, 26) [4], *Ctla4* (22 ± 11% increase in combined clusters 7, 12, 19, 26) [63], *Tigit* (153 ± 71% increase in combined clusters 7, 12, 19) [64, 65] and *Havcr2/TIM3* (35 ± 13% increase in cluster 7) [3, 63] all exhibited small but significantly increased expression in ICs from *Shb* iECKO tumors (Fig. 2D). *Ctla4*, *Tigit*, and *Havcr2/TIM3* are expressed at high levels in exhausted T cells [38]. *Cd200* (23 ± 11% decrease in combined clusters 7, 12, and 26), a marker for active T effector cells [66, 67], and *Cd80* (14 ± 4% decrease in combined clusters 12 and 26) [68], which when expressed in antigen‐presenting cells competes with the *CTLA4* gene product for CD28 binding on T cells, were decreased under the same conditions. The expression levels of *Lag3*, *Cd274* (PD‐L1), *Pdcd1lg2* (PD‐L2), or *Pdcd1* (PD1) [63] were unchanged. Although PD‐L1 and PD1 are important immune checkpoint proteins, their lack of altered expression does not necessarily invalidate the relevance of the other immune checkpoint gene expression changes since the final immunosuppressive state will be the product of many modest changes that in the end will tip the response towards a pro‐tumoral state.

Taken together, the IC gene expression profile in response to *Shb* iECKO, despite moderate changes, suggests an overall immunosuppressive mode, which is possibly a consequence of the altered chemokine and cytokine profile observed in myeloid cells. This view is further reinforced by the increased cell number of IFN‐macrophage cluster 4, which corresponds to a *Cd274* (PD‐L1)‐high macrophage cluster commonly present in tumors, thus likely to be tumor supportive, and the reduction in cell number of the antigen‐presenting cDC1 cluster 25 [37].

### 3.3 Gene ontology (GO) analysis of IC DEGs as a consequence of EC *Shb* deficiency

GO molecular function by Toppgene (https://toppgene.cchmc.org/enrichment.jsp) pertaining to ‘structural constituent of ribosome’ (*P* < 0.0001), ‘CCR1, CCR2, CCR4 and CCR5 chemokine receptor binding’ and ‘cytokine receptor binding’ (*P* < 0.02), ‘cell adhesion molecule binding’ (*P* < 0.02) and ‘lipoprotein particle binding’ (*P* < 0.02) were observed in macrophages/monocytes (Fig. 2E). ‘Thioredoxin peroxidase activity’ (*P* < 0.01) was observed in cluster 18. These conferred significant changes to the biological processes ‘response to virus and biotic stimulus’, ‘response to lipid’, ‘regulation of immune response’, and ‘lymphocyte or T cell activation’. As cellular components, ‘ribosome’ and ‘focal adhesion’ were identified. In T cells, ‘chemokine receptor binding’ (*P* < 0.0001), ‘antigen binding’ (*P* < 0.05), ‘collagen binding’ (*P* < 0.05) and ‘immune receptor activity’ (*P* < 0.05) were observed (Fig. 2E). These had implications for ‘regulation of immune system process’, ‘response to biotic or virus stimulus’ and ‘T cell activation’. Cellular components of ‘endolysosome’ (*P* < 0.02) and ‘extracellular matrix’ (*P* < 0.05) were changed. DCs (cluster 25) exhibited ‘structural constituent of ribosome’ (*P* < 0.05) and ‘response to cytokine’ (*P* < 0.05). Changed biological processes were ‘response to virus or biotic stimulus’ and ‘regulation of immune system process’ (Fig. 2E). Table S4 provides a detailed list of GOs in individual clusters or in merged macrophage/monocyte, T cell and DC populations. The changes are in line with properties that infer an altered immune response.

### 3.4 EC gene expression changes in *Shb*‐deficient tumor

**Fig. 3:**
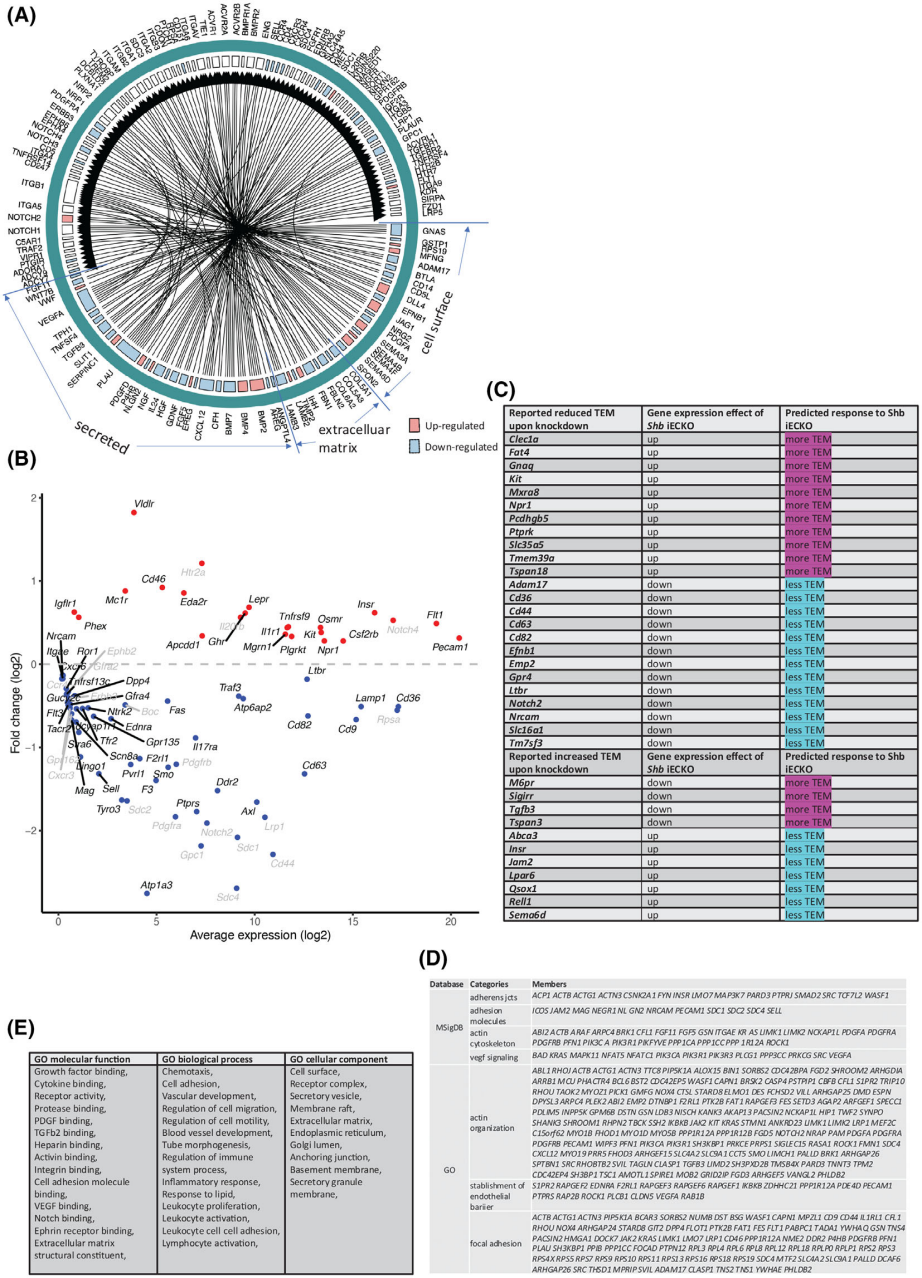
Breast cancer EC gene expression changes upon *Shb* iECKO. (A) Wheel chart showing EC ligand gene expression changes according to published ligand–receptor interactions and cognate receptors expressed in EC. Ligands were categorized as secreted proteins, cell surface proteins, and extracellular matrix proteins. Red = increased expression, blue = decreased expression. (B) Volcano plot of the EC receptor gene expression changes according to published ligand–receptor interactions. Receptors with intracellular ligands have been omitted. Genes with gray text represent EC receptor genes differentially expressed with cognate ligands significantly differentially expressed in EC. (C) Current EC gene expression changes and described effects upon knockdown in EC on leukocyte transendothelial migration (TEM) [69]. (D) Significant EC gene expression changes after *Shb* iECKO grouped according to GSEA mSigDB (adherens junctions, adhesion molecules, actin cytoskeleton, VEGF signaling) and GO (actin organization, establishment of endothelial barrier, focal adhesion). (E) GO categories of significant EC gene expression changes, EC, endothelial cells; GO, gene ontology; iECKO, conditional deletion of *Shb* in EC. Significant changes in A–E were determined on 11 wild‐type and 10 iECKO samples performed on four separate occasions using a paired Student's *t*‐test.

Bulk EC RNAseq was performed on wild type and *Shb* iECKO samples in four separate experiments from nine wild type and eight KO tumors. Means of each genotype in each separate experiment were calculated and compared by paired Student's *t*‐test. Significant differences in gene expression and expression values for all genes are shown in Table S5A,B. Gene ontology (GO) analysis for the differentially expressed genes is shown in Table S6. The GOs recorded were fairly non‐specific, including a number of processes and components such as the cytoskeleton, focal adhesions, ribosomes, endothelial barrier, and apoptosis. Previous literature has compiled a large number of ligand/receptor interactions [32] and using these, we then analyzed the significant EC gene expression changes in a ligand/receptor context. EC ligand changes (Table S5C) and their EC receptor partners are shown in Fig. 3A. The EC ligands fall into the categories ‘secreted ligand, cell surface ligand (including intracellular modification of ligand signaling) and extracellular matrix’. Finally, all significant EC receptor changes are displayed in Fig. 3B, Table S5C. To understand the possible relevance of the differentially expressed EC ligand and EC receptor interactions in Fig. 3A,B and Table S5, the literature was searched for reported responses, and the most likely and prominent effects in relation to the vasculature and its influence on immune cells were presented (Table 1). Gene expression changes that support decreased sprouting angiogenesis and leakage according to the literature strongly predominate, falling in line with the experimental observations. These include *Notch4*, *Dll4*, *Jag1*, *Src*, *Prprj*, *Sdc1*, *Sdc2*, *Sdc4*, *Vegfa*, *Fgd5*, *Ptk2b*, *Efnb1*, *Sema3a*, *Sema4b*, and *Sema6d*. Only an increase in *Plcg1* stands out as a change suggesting an opposite effect. There were numerous EC DEGs coding for receptors (Fig. 3B, Table S5) of possible interest. *Erbb3* has been shown to be important for the endothelial cytoskeleton *in vitro* [112]. Additionally, *Ednra* codes for an endothelin receptor, *Gpr135* a bradykinin receptor, *Npr1* a natriuretic receptor, *Ptprs* a receptor tyrosine phosphatase that localizes to junctions, *Rpsa* a laminin receptor, and *Traf3* a product that interacts with *Cd40lg* on T cells according to Genbank. These have not been explored in detail in the present context but are potentially relevant mediators in immune responses.

**Table 1:** EC gene expression changes and their potential impact on the vasculature and immune system. DEG, differential gene expression; EC, endothelial cell; IC, immune cell; iECKO, EC deletion; M1, macrophage type 1; MDSC, myeloid‐derived suppressor cell; Teff, T effector cell; TEM, trans endothelial migration; Th, T helper; Treg, T regulatory cell.

Endothelial DEGs by total RNAseq	Changes in response to *Shb* iECKO	Predicted outcome
*Notch4, Dll4, Jag1, Hes1, Hey1, Flt1, Shroom2*	Increased expression	Reduced sprouting angiogenes [70, 71, 72]
*Plcg1*	Increased expression	Increased angiogenesis [73]
*Src, Wnt7b, Ptprj (Dep1), Sdc1, Sdc2, Sdc4, Vegfa, Cxcl12*	Decreased expression	Reduced angiogenesis [74, 75, 76, 77, 78, 79, 80, 81, 82]
*Pdgfd, Flt1, Fgd5, Ptprk, Sema3a*	Increased expression	Reduced leakage [72, 83, 84, 85, 86]
*Ptprj (Dep1), Src, Ptk2b (Pyk2), Efnb1, Sdc4, Vegfa, Sema4b, Sema6d*	Decreased expression	Reduced leakage [77, 78, 82, 87, 88, 89]
*Plcg1*	Increased expression	Increased leakage [73]
*Pecam1, Fgd5, Piezo2*	Increased expression	Increased IC TEM [22, 86, 90], Piezo1 increased IC TEM [69]
*Cxcl12, Hgf*	Decreased expression	Decreased IC TEM or recruitment [80, 91]
*Jam2*	Increased expression	Decreased IC TEM [69]
*Bmp4, Cxcl12*	Increased expression	Reduced T cell activation [80, 92]
*Pdgfd*	Increased expression	Increased monocyte TEM [85]
*Sema3a*	Increased expression	Increased *Tgfb1* expressing monocyte TEM [83]
*Vwf*	Increased expression	Increased platelet and monocyte adhesion [93]
*Dll4, Jag1*	Increased expression	Increased Th1, Th2 and Treg differentiation, increased MDSC infiltration and M1 polarization [94, 95]
*Icosl, Icos*	Decreased expression	Reduced Treg and Teff TEM [96, 97] and interactions with other immune cells [98]
*Cd44*	Decreased expression	Reduced lymphocyte TEM [99]
*Cd63, Sell, Ccr4*	Decreased expression	Reduced IC capture [22, 99, 100]
*Cd82, Ltbr*	Decreased expression	Reduced IC TEM [101, 102]
*Cd83*	Decreased expression	Reduced antigen presentation [103]
*Cd86*	Decreased expression	Decreased immune checkpoint interaction by competition with Ctla4 [68]
*Bmp7, Ptk2b (Pyk2), Axl, Sell, Efnb1, Tnfsf4*	Decreased expression	Less IC adhesion and TEM [88, 104, 105, 106]
*H2‐Q4, H2‐Q6, H2‐Q7 (class II MHC)*	Increased expression	Increased antigen presentation, increased Treg transmigration, EC‐EC interaction [107, 108, 109]
*Sema4b*	Decreased expression	Increased Treg infiltration [110]
*Cxcl12*	Decreased expression	Reduced inflammation and immunosuppression [80, 111]
*Pik3c2b, Pik3r1, Pik3r3, Pik3r4*	Increased expression	Pleiotropic effects, increased PI3'‐kinase activity

A comparison of the present data with those of Wang et al. [69] on genes relevant for leukocyte transendothelial migration (TEM) has been compiled in Fig. 3C. The data demonstrate disparate effects with respect to predicted TEM responses, further supporting pleiotropic effects of EC *Shb* deficiency on the immune system. EC gene expression changes pertaining to adherens junctions (https://www.gsea‐msigdb.org/gsea/msigdb/human/geneset/KEGG_ADHERENS_JUNCTION.html), adhesion molecules (https://www.gsea‐msigdb.org/gsea/msigdb/human/geneset/KEGG_CELL_ADHESION_MOLECULES_CAMS.html), actin cytoskeleton (https://www.gsea‐msigdb.org/gsea/msigdb/human/geneset/WP_REGULATION_OF_ACTIN_CYTOSKELETON.html) and Vegfa signaling (https://www.gsea‐msigdb.org/gsea/msigdb/human/geneset/KEGG_VEGF_SIGNALING_PATHWAY.html) are displayed in Fig. 3D. In addition, gene expression changes pertaining to the GO categories ‘actin organization’, ‘establishment of endothelial barrier’, and ‘focal adhesion’ have been indicated (Fig. 3D), implicating their importance for vascular and immune cell responses. Prominent GOs based on DEGs presented in Fig. 3A,B are displayed in Fig. 3E.

In summary, the observed reduced angiogenesis and leakage phenotypes [29] are easily supported by the EC gene expression changes and are in line with ‘vascular normalization’. Of further importance are the components adherence junctions, actin organization, and extracellular matrix. In addition, numerous gene expression changes were observed that are likely to influence the immune system in a complex manner. The changes may allow for an immune cell environment to evolve that shows important changes in cytokines, chemokines, cell adhesion molecules, and immune checkpoint proteins.

### 3.5 EC and IC DEGs and their interactions

**Fig. 4:**
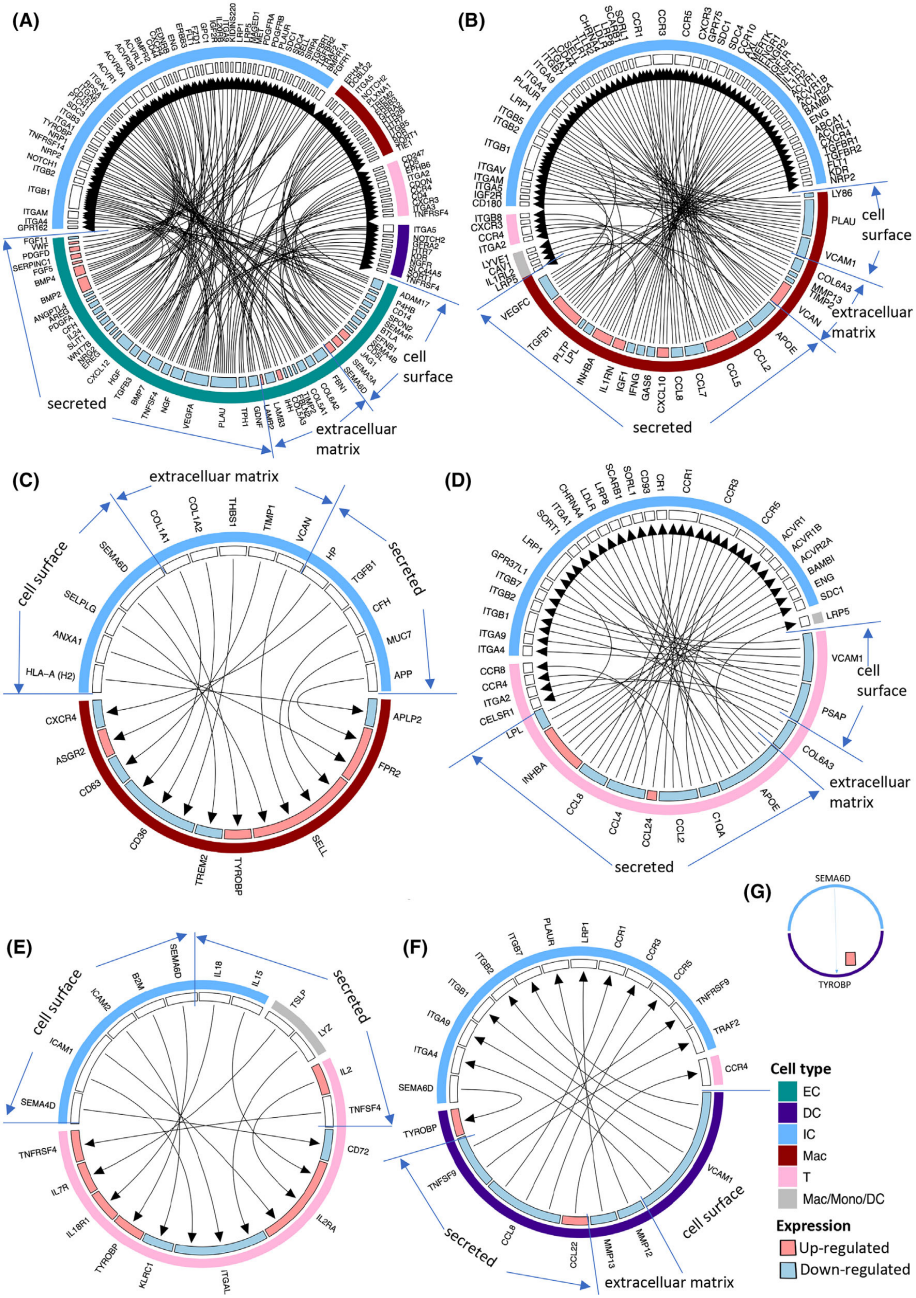
EC‐IC interactions according to published ligand–receptor interactions based on tumor EC and IC gene expression changes in *Shb* iECKO mice. (A) Wheel chart of EC ligand (secreted, cell surface, extracellular matrix) and potential cognate receptors expressed in ICs (monocytes/macrophages, T cells, DCs, or ICs in general according to color code). (B) Monocyte/macrophage ligand gene expression changes and potential cognate receptors in T cells, monocytes/macrophages/DCs or ICs in general. (C) Monocyte/macrophage receptor gene expression changes with potential cognate ligands in ICs in general. (D) T cell ligand gene expression changes and potential cognate receptor gene expression changes in T cells, monocytes/macrophages/DCs and ICs in general. (E) T cell receptor gene expression changes and cognate ligands in T cells, monocytes/macrophages/DCs and ICs in general. (F) DC ligand gene expression changes and cognate receptors in T cells or ICs in general. (G) DC receptor gene expression changes and cognate ligand in ICs in general. Increased (red) or decreased (blue) expression has been color coded in A–G. DC, dendritic cells; EC, endothelial cells; IC, immune cells; iECKO, conditional deletion of *Shb* in EC; Mac, macrophages; Mono, monocytes; T, T cells. *N* = 6 for each IC genotype and *n* = 11 for wild‐type and *n* = 10 for iECKO genotype. Statistical differences were determined as in Figs 2 and 3.

The potential interactions between EC DEG ligands and cognate receptors on ICs are depicted in Fig. 4A. A number of ligand/receptor pairs are likely to affect IC function. Concerning EC/IC interactions, that is, IC adhesion to EC, TEM, and IC activation by ECs, a disparate pattern of responses was observed (Table 1). This provides a rationale for the overall pro‐tumoral/immunosuppressive response since it may cause selective recruitment of relevant immunosuppressive cell populations. One response that has previously been characterized is the recruitment of *Tgfb1*‐expressing monocytes by endothelial *Sema3a* [83]. *Tgfb1* codes for an immunosuppressive cytokine that exhibited elevated gene expression in a macrophage cluster, and this could reflect increased *Sema3a*‐dependent monocyte TEM that has subverted the immune system to a pro‐tumoral phenotype. However, numerous other changes which have a potential impact on leucocyte capture, recruitment, TEM, and activation, thus contributing to the immune environment, are apparent (Table 1). Those include *Efnb1*, *Dll4/Jag1*, *Cxcl12*, *Pdgfa/Pdgfd*, *Sema4b*, *Tnfsf4*, and *Bmp7* with respect to EC ligand changes. *Cxcl12*, *Dll4*, *Jag1*, *Pdgfd*, and Class II MHC appear to exhibit pleiotropic effects in this context (Table 1). *Areg* (Fig. 3A) has been shown to promote an inflammatory environment [113] and thus a reduction in *Areg* expression is compatible with reduced inflammation. *Bmp4*, *Hgf*, *Sema3a*, *Sema6d*, *Vegfa*, and *Wnt7b* have been reported to exert diverse direct or indirect effects on IC (Table 1). *Btla*, when expressed in T cells, suppresses immune responses [114]; *Ihh* is required for thymus development [115] and *Il24* partakes in inflammation [116]. *Ngf* exerts multiple roles in the immune system [117] and *Tgfb3* is both immunosuppressive and immunostimulatory depending on context [118]. *Vwf* plays a role in leukocyte recruitment [119].

In summary, EC gene expression changes are likely to exert pleiotropic effects on the immune system. One alteration that may partially explain the anti‐inflammatory status of the *Shb‐*KO tumors is *Sema3a*‐dependent recruitment of *Tgfb1‐expressing* monocytes.

Macrophage/monocyte ligand gene expression changes that may interact with IC receptors are shown in Fig. 4B. Many of these have already been described above (Fig. 2B) as macrophage/monocyte DEGs. *Plau* and *Timp2* are also present among the EC DEGs. *Gas6* stimulates the EC DEG receptors *Axl* and *Tyro3*, suggesting reciprocal IC to EC signaling. A reduction in macrophage‐derived *Vegfc* will reduce lymphangiogenesis [120] whereas *Il1rn* is anti‐inflammatory [121]. *Apoe* (apolipoprotein E), *Lpl* (lipoprotein lipase), and *Pltp* (phospholipid transfer protein) pertain to aspects of lipid metabolism.

Differentially expressed macrophage/monocyte receptors were *Aplp2*, *Fpr2*, *Sell*, *Tyrobp*, *Trem2*, *Cd36*, *Asgr2*, and *Cxcr4* (Fig. 4C). *Trem2* has been described above as an immune checkpoint protein, and *Sell* and *Cd36* were also observed among EC DEGs. *Cxcr4* is the *Cxcl12* receptor, and endothelial *Cxcl12* was reduced, suggesting another example of synergy between EC and IC signaling.

T cell ligand gene expression changes that may interact with IC receptors are shown in Fig. 4D. These largely overlapped with the macrophage/monocyte changes except for *Ccl4*, *Ccl24*, and *Psap* (prosaposin). Differentially expressed receptor genes were *Cd72*, *Il2ra* (CD25), *Il7ra*, *Il18r1*, *Tnfrsf4*, *Itgal*, *Tyrobp*, and *Klrc1* (killer cell lectin‐like receptor C1) (Fig. 4E). Particularly, CD25 is an important player in the immunosuppressive role of Tregs.

DC ligand gene expression changes that may interact with IC receptors and DC receptors that interact with IC ligands are shown in Fig. 4F,G. Besides *Ccl22*, which has been discussed above, the other changes overlap largely with macrophage gene expression changes.

### 3.6 Vascular barrier morphology and tumor hypoxia as a consequence of EC *Shb* deficiency

**Fig. 5:**
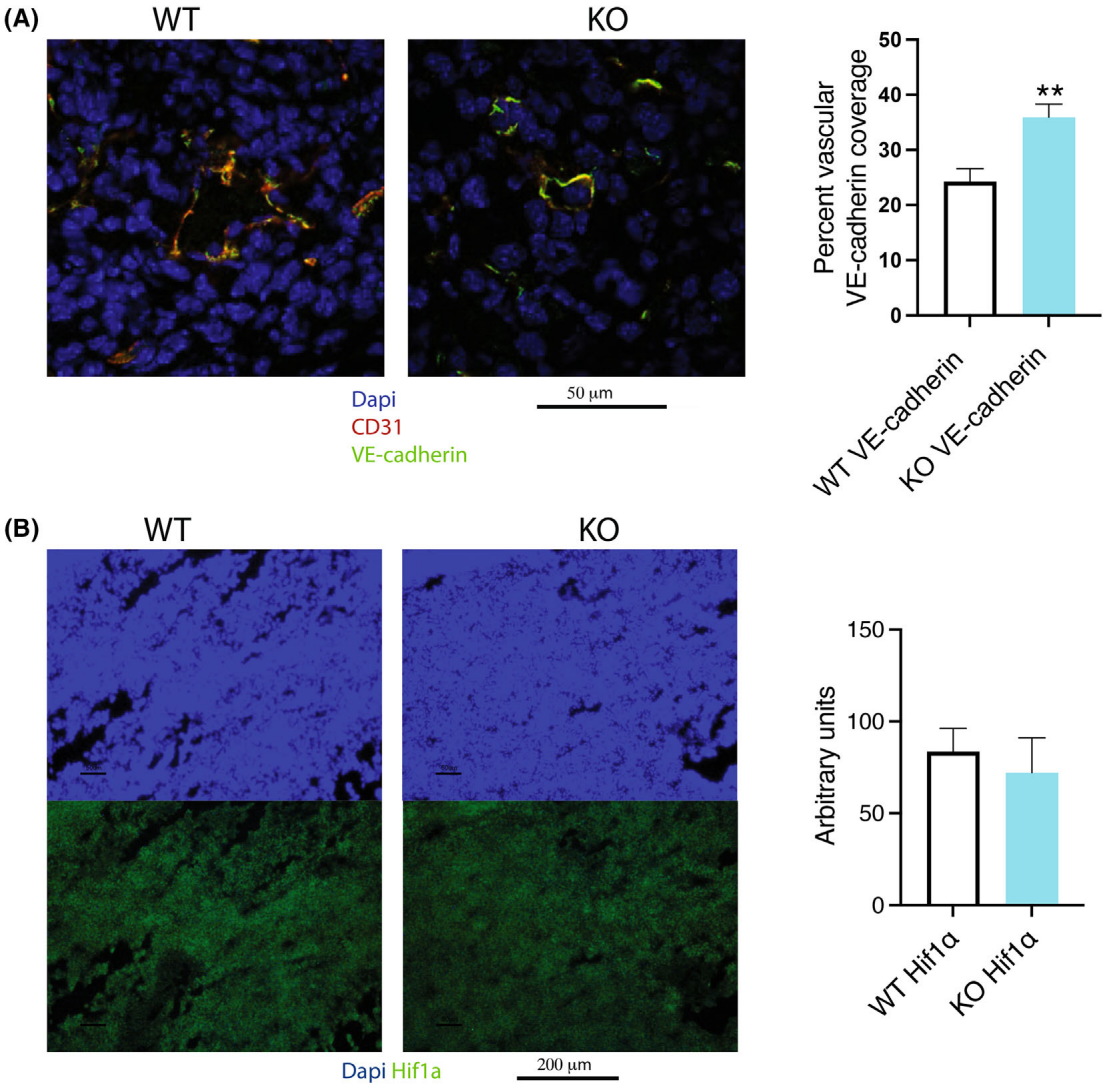
E0771 tumors in mice in wild‐type (WT) or *Shb* iECKO (KO) mice. (A) Staining for CD31 (red), VE‐cadherin (green), and DAPI (blue). Vessels with partial or nearly complete VE‐cadherin coverage are shown by confocal microscopy. Quantitation for 3 WT and 5 KO separate mice (percent coverage of vascular circumference ± SEM) is shown. ** indicates *P* < 0.01 by Student's *t*‐test. (B) HIF1a (hypoxia‐induced factor 1a) staining of wild‐type (WT) and *Shb* iECKO (KO) tumors with quantitation (normalized unit intensity ± SEM) for 3 WT and 5 KO separate mice. iECKO, conditional deletion of *Shb* in EC. Scale bars for A (50 μm) and B (200 μm) have been indicated.

Of interest to the immune cell gene expression profile in the breast carcinoma model of *Shb* iECKO is the function of the vasculature with respect to leakage and oxygenation. We previously reported less leakage in this model [29]. This is confirmed by more extensive VE‐cadherin coverage of the tumor vasculature as a consequence of *Shb* iECKO (Fig. 5A), in agreement with reduced leakage and further suggesting that the immune phenotype is not a direct consequence of increased vascular leakage. In addition, there were no signs of increased hypoxia as assessed by *Hif1a* staining (Fig. 5B), contradicting the assumption that the immune cell alterations were hypoxia‐induced and further supporting the notion of vascular normalization.

### 3.7 Vascular and immune cell characteristics of human breast cancer

In relating the experimental mouse data of increased metastasis and less leakage paralleled by an immunosuppressive environment to human breast cancer, a cohort of 20 triple‐negative breast cancers were stained for vascular and immune cell markers. The tumor characteristics have been summarized in Table S7. Sections were stained for VE‐cadherin (endothelial adherens junctions), FpA (fibrinogen peptide A reflecting leakage, [122]), CD4 (T helper cells), FOXP3 (Tregs), CTLA4 (immune suppressing T cells expressing the CTLA4 immune checkpoint protein), CD8 (T killer cells), PD1 (immune suppressed T cells responding to PD‐L1), PD‐L1 (*CD274* which is an immune checkpoint protein), CD163 (*CD163* which is a marker for macrophages), CD20 (*MS4A1* which is a marker for B cells), and granzyme B (*GZMB* which is a marker for T and NK cells active in target cell killing). The stroma surrounding the tumor proper was commonly rich in immune cells and displayed a higher vascular density (Fig. S3A,B).

**Fig. 6:**
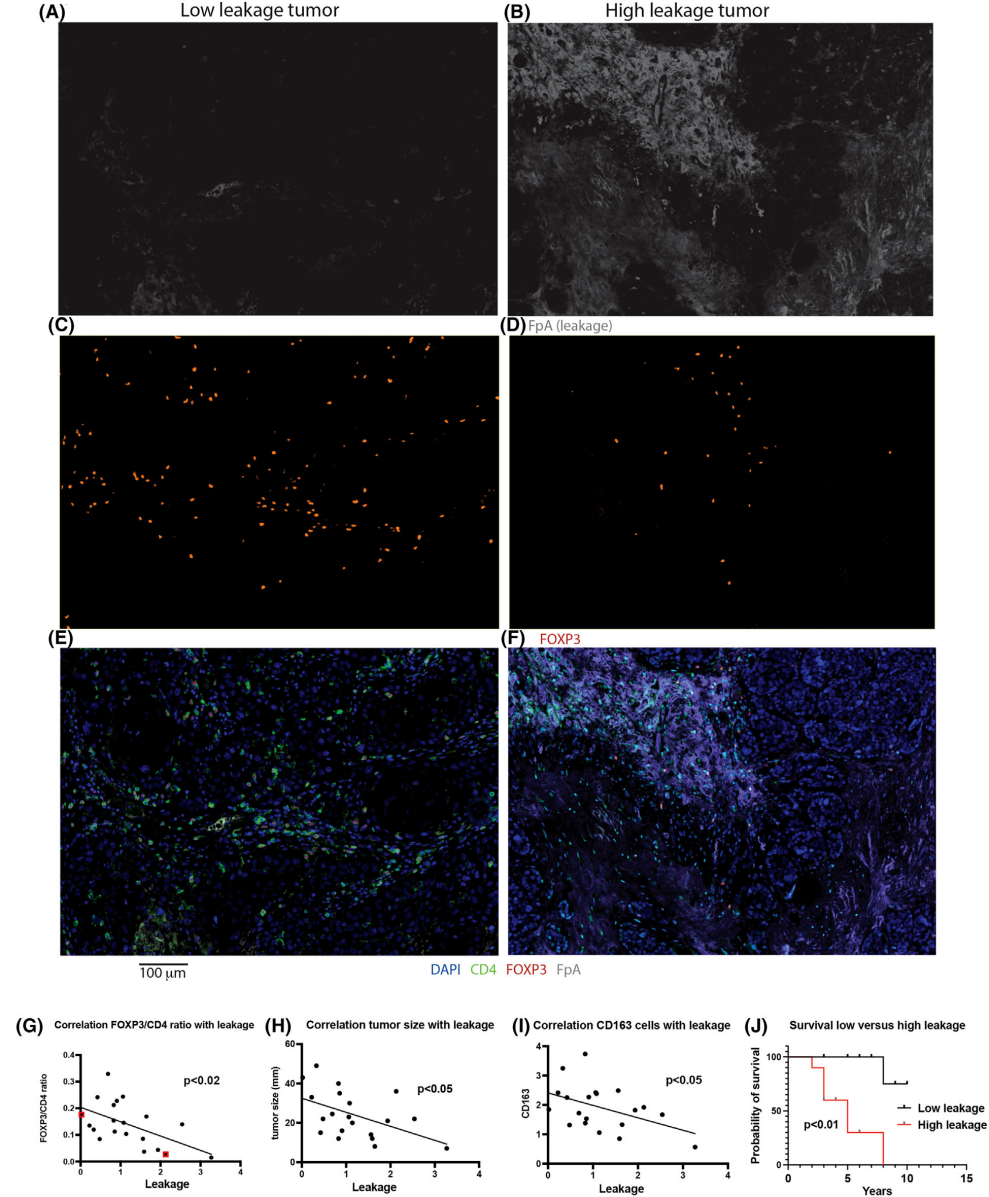
Human tumor IC and leakage staining. One tumor with low leakage (A, C, E) and one with high leakage (B, D, F) are shown. FpA (leakage A–B; gray), FOXP3 (Tregs C–D; red) and merged image (E, F) with CD4 (T helper cells; green) and DAPI (blue) in addition are shown. (G) Negative correlation between the FOXP3/CD4 ratio (indicating the relative fraction of Tregs) and leakage is shown (*P* < 0.02). The two tumors in panels A–F are indicated with red boxes. (H) Negative correlation between leakage and tumor size (diameter in mm). *P* < 0.05. (I) Negative correlation between leakage and CD163^+^ cells. *P* < 0.05. (J) Kaplan–Meier survival plots of overall survival in years among patients with lower or higher than median leakage. *P* < 0.01 by log‐rank sum test. Alive patients at the end of the study were censored. Corresponding cox regression for overall survival comparing patients with leakage above versus below median showed a hazard ratio of 13.69 (95% CI: 1.62–115.61, *P* = 0.016, Wald test). Cells per unit area (0.002 mm^2^) were determined as well as FpA‐positive area in percent. Significant Pearson *r* correlation *P* values are indicated. Correlation coefficients (*r*) in G–I are −0.540, −0.491, and −0.453, respectively. IC, immune cells. *N* = 20 patients for A–J. Scale bar of 100 μm for A–F is shown below.

Tumor staining for FpA, FOXP3, CD4, and DAPI is shown in Fig. 6A–F. Among the immune cell populations in the different tumors, FOXP3‐stained nuclei in almost exclusively CD4^+^‐cells (Fig. 6C,E), and tumor FOXP3^+^ cell counts correlated with those of CD4^+^ cells, indeed supporting the notion that these are primarily CD4^+^ Tregs (Fig. S4A). The ratio FOXP3^+^/CD4^+^ was thus taken as the relative proportion of Tregs among T helper cells. CTLA4^+^ correlated with CD4^+^ cells and FOXP3^+^/CD4^+^ cells (Fig. S4B,C), suggesting that Tregs commonly express the immune checkpoint protein CTLA4. However, CTLA4 staining occasionally was observed in non‐immune cells, and this was particularly apparent in one tumor (Fig. S4L, tumor from patient six), suggesting that some tumor cells also express CTLA4 in this cohort. CD4^+^ cells also correlated positively with CD8^+^, CD20^+^, and CD163^+^ cells (results not shown), in line with an overall inflamed tumor phenotype. Granzyme B was determined as a ratio of GZMB^+^/CD8^+^ cells, and this ratio did not correlate with any other vascular or IC marker among the tumors (results not shown). PD1, a marker for exhausted CD8^+^ cells, also showed no correlation with any other vascular or IC marker (results not shown). The percentages of GZMB^+^/CD8^+^ or PD1^+^/CD8^+^ cells were low (never exceeding 21% in any tumor), and only granzyme B showed relevant co‐expression with CD8 (Fig. S3C,D), suggesting that the tumors show no propensity toward active cytotoxicity or CD8^+^ exhaustion.

Tumor diversity with respect to the vascular and IC markers was extensive (Table 2). It can be noted that tumors 6, 9, and 11 showed high numbers of cells expressing the immunosuppressive markers FOXP3^+^/CD4^+^ and CTLA4^+^. These tumors did not display high levels of leakage. On the other hand, tumors 1, 2, and 14 showed low numbers of cells expressing these immunosuppressive markers while simultaneously exhibiting high leakage. To better understand tumor diversity with respect to vascular and IC markers, correlations between the variables were determined.

**Table 2:** Tumor characteristics with respect to vascular and immune cell markers and metastasis. +, indicates more than the median; −, less than the median.

Tumor	FpA	VE‐cadherin	CD4	FOXP3/CD4	CTLA4	GZMB/CD8	PD1/CD8	CD8	PD‐L1	CD20	CD163	Metastasis
3	+	+	−	+	−	+	+	−	−	+	−	−
4	−	−	−	+	−	+	+	−	+	−	+	−
6	−	−	−	+	+	−	−	−	−	−	−	−
7	−	−	−	+	−	+	+	−	−	−	−	−
8	−	+	−	−	−	−	−	−	−	−	−	−
9	−	+	+	+	+	−	+	+	+	+	+	−
11	−	−	+	+	+	−	+	+	+	+	+	−
14	+	+	+	−	−	−	−	+	−	+	+	−
15	−	−	−	−	−	−	−	−	−	+	−	−
18	+	+	+	+	+	+	−	+	+	+	+	−
1	+	−	−	−	−	−	+	−	−	−	−	+
2	+	−	−	−	−	+	+	−	−	−	−	+
5	+	+	+	−	+	+	−	+	+	−	+	+
10	+	+	−	−	−	−	−	−	−	−	−	+
12	+	+	+	+	+	−	+	−	+	+	−	+
13	−	−	+	−	+	+	+	+	+	+	+	+
16	+	−	−	−	−	+	−	+	−	+	−	+
17	−	+	+	+	+	−	−	+	+	−	+	+
19	−	−	+	−	+	+	−	+	+	−	+	+
20	+	+	+	+	+	+	+	+	+	+	+	+

When comparing vascular density (VE‐cadherin staining) with immune cell populations in these tumors, a positive correlation was observed for CD4 (Fig. S4D), FOXP3 (Fig. S4E), and CTLA4 (Fig. S4F). This suggests that a high vascular density indeed promotes immune cell infiltration. However, leakage did not correlate with vascular density and tumor infiltration of CD4^+^, FOXP3^+^, CTLA4^+^, and PD‐L1^+^ cells. The only T cell population that showed a correlation (negative) with leakage was FOXP3^+^/CD4^+^ cells, that is, the relative Treg abundance, which was significantly reduced in tumors with high leakage (Fig. 6A–G). The relative Treg abundance may be a significant factor in immune suppression since not only total cell population numbers matter but also the relative proportion of the particular cell population. Tumor size and CD163 (a marker for macrophages) also displayed a negative correlation with leakage (Fig. 6H,I), suggesting a possible negative relationship between leakage and tumor growth—macrophages—Tregs. The immune checkpoint protein PD‐L1 was largely expressed in CD163^+^ cells (Fig. S3E,F) and correlated with CD4^+^ and CD163^+^ expression (Fig. S4G,H).

In contrast to this, tumor metastasis appeared to be less common in patients with tumors exhibiting less leakage. Whereas seven of 10 high leakage patients exhibited metastasis (Table 2), metastasis in patients with low leakage was observed in three of 10 patients. This difference was, however, not statistically significant (*P* = 0.074 by chi‐square test), likely due to the low number of patients. However, survival was increased in patients with low leakage (Fig. 6J). There was no significant difference in age between the ‘high’ versus ‘low’ leakage groups (65.8 ± 4.3 and 68.4 ± 4.6 years, respectively, *P* = 0.69).

The findings partly confirm those of the experimental mouse model of *Shb*‐gene inactivation in ECs that resulted in reduced tumor leakage and increased immune suppression. However, vascular leakage appears to play a predominant role over immune suppression in human breast cancer metastasis.

## 4 Discussion

This study was conducted in order to understand the role of ECs in the regulation of breast cancer immune responses by investigating IC and EC gene expression changes under conditions of low vascular leakage. This was motivated by the observation that in an experimental mouse model with conditional *Shb* deficiency in ECs, orthotopic E0771.lmb triple‐negative breast carcinoma metastasis was increased due to immune suppression and not to increased vascular leakage [29]. The main findings of this study on human breast cancer are that immune suppression is associated with reduced vascular leakage, whereas metastasis may be more prevalent in tumors with high leakage. The experimental mouse model suggests that this was due to a primary endothelial effect since *Shb* deficiency was induced in endothelial cells. This could infer that the human data on immune suppression and leakage also reflect a primary endothelial alteration.

In‐depth analysis of the gene expression changes occurring in EC as a consequence of *Shb* deficiency mostly supports a non‐leaky vasculature, as does the increased fraction of vascular VE‐cadherin coverage. This vascular phenotype, together with unchanged tumor oxygenation, is in line with the concept of ‘vascular normalization’ [12]. A recent study reported that vascular normalization by angiogenesis inhibition selectively increased CD8^+^ T cell infiltration into hepatocellular carcinoma [125]. That finding agrees with our present finding of reduced vascular leakage supporting selective extravasation of a specific immune cell population. However, in contrast to the present findings, an IC component (CD8^+^ effector cells) that promoted an immune response was selectively stimulated in that study. Such a dichotomy suggests that a specific IC response to ‘vascular normalization’ is subject to control by the local tumor milieu, including the degree of oxygenation and/or cytokine/chemokine production, and consequently the responses cannot easily be categorized as simply ‘immune suppressive’ or ‘immune stimulatory’ but rather a mix of these in which the balance may be skewed in either direction due to minor local differences. Although vascular normalization has, in many cancer models, been found to improve the efficacy of immunotherapy [126, 127], we do not think that our present data necessarily contradicts this notion. An immunosuppressed environment with an increased abundance of Tregs, as currently observed, may be more amenable to immune checkpoint inhibition since this cell type responds to anti‐PD‐L1 immunotherapy [128].

It is uncertain whether the selective immune response is solely a consequence of reduced vascular leakage or reflects a parallel endothelial process. Numerous EC gene expression changes were observed occurring in the non‐leaky *Shb* iECKO tumors, and these would be expected to exert pleiotropic effects on IC function. This provides a rationale for the disparate immune responses in different tumors—depending on local conditions, different responses will predominate.

The IC landscape in the experimental model suggests a primarily immunosuppressive environment with relatively more Tregs, slightly increased immune checkpoint protein expression, and fewer cDC1 antigen‐presenting cells. Whether this is a direct consequence of the EC phenotype or secondary to EC‐dependent changes in the monocyte/macrophage populations cannot presently be determined. Among the monocyte/macrophage populations, a large plethora of changes was observed, some immune inhibitory and others immune stimulatory, making it difficult to predict the changes that are responsible for immunosuppression.

When a human triple‐negative breast cancer cohort was investigated, a considerable variation between different tumors with respect to vascular and IC characteristics was noted. Conspicuously, three tumors with high leakage showed low infiltration of immune suppressive cells, whereas three tumors with low leakage had high infiltration of immune suppressive cells. Thus, it was considered that determining correlations between different parameters within the cohort was an appropriate manner in order to establish relevant differences. Accordingly, a negative correlation between leakage and Tregs or CD163 macrophages was observed. That both were similarly altered is in agreement with an interdependence between Tregs and myeloid cells. However, the presence of Tregs in human breast cancer appears to play a subordinate role compared with leakage for metastasis since tumors with less leakage did not exhibit much metastasis but rather longer survival. This is in contrast to the mouse experimental model. Although experimental mouse models are useful in proposing mechanistic aspects of relevance to human disease, their applicability to full‐scale understanding is limited. Unlike metastasis, tumor size was increased in tumors with low leakage in human disease. This indicates that tumor growth and tumor metastasis are two independent processes.

It should be noted that the tumor sample size is limited (20 patients) and thus future validation in larger, independent tumor cohorts is required.

## 5 Conclusions

**Fig. 7:**
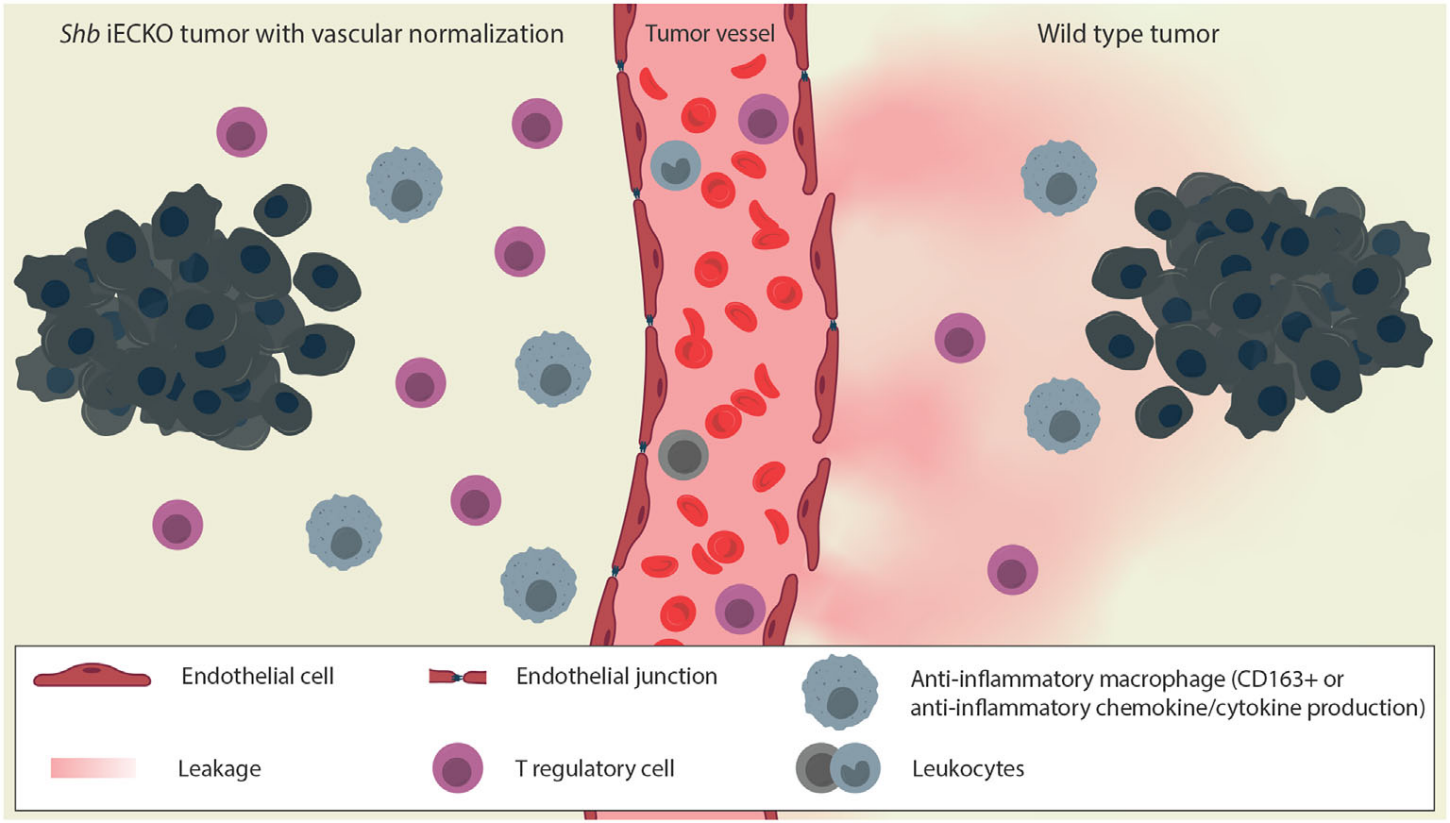
Schematic figure illustrating the differences between wild‐type breast tumors (right side) and tumors with ‘vascular normalization’ as observed in *Shb* iECKO mice (left side). The wild‐type tumors have high leakage, relatively few Tregs, and few anti‐inflammatory macrophages (defined as CD163 positive [123, 124] and/or with altered chemokine/cytokine profiles). The *Shb* iECKO phenotype displays low leakage in line with ‘vascular normalization’ and has more Tregs and anti‐inflammatory macrophages. The human breast cohort displays variability with respect to these alternate states, giving rise to distinct tumor characteristics. EC, endothelial cell; iECKO, conditional deletion of *Shb* in EC; Treg, T regulatory cell.

In summary, the EC phenotype influences the immune response, which in the case of breast carcinoma is primarily immune suppressive in tumors with low vascular leakage (Fig. 7). A likely explanation is selective IC extravasation which alters the tumor IC landscape, thus conferring numerous gene expression changes including those of chemokines. Consequently, there is a selective increase of Tregs in tumors with low vascular leakage. From a general perspective, the data are in line with the notion that selective IC extravasation is dependent on intact functional junctions and that immunosuppressive or immunostimulatory conditions are the consequence of the local environment. This suggests that the vasculature in human tumors plays a crucial role in shaping immune dynamics and therefore is an important target of therapeutical intervention. Targeting vascular function could either enhance antitumor immune responses or limit metastatic spread, depending on the specific characteristics of the tumor microenvironment.

## Conflict of interest

The authors declare no conflict of interest.

## Author contributions

LH performed bioinformatic analysis and figure design. CT conducted the tumor experiments and CD45^+^/CD31^+^ cell isolations. NH performed multiplex staining and scanning of human tumor samples. AB did mouse tumor staining and participated in tumor experiments. AS characterized breast cancer patients. EN helped analyze vascular staining patterns. MP contributed to analyzing IC gene expression changes and supported the study. CS designed the human breast cancer study. LH, CT, CS, and MW all participated in study design and interpretation of data. MW wrote the paper. All authors have read and commented on the manuscript and approved submission.

## Supporting information


**Fig. S1.** Cluster expression of signature macrophage and monocyte gene markers.
**Fig. S2.** Cluster expression of signature T cell and DC (dendritic cell) gene markers.
**Fig. S3.** (A) Typical staining for VE‐cadherin (vascular, red), FpA (leakage, gray), and CD4^+^‐cells (green). (B) DAPI of the same section. Tumor boundary has been indicated. (C) CD8 (white) and GZMB (green) or (D) PD1 (red). (E, F) CD163 (yellow) and PD‐L1 (red) in human breast cancer stroma.
**Fig. S4.** Correlations between CD4 and FOXP3 (A), CTLA4 and CD4 (B) and CTLA4 and FOXP3/CD4 (C) in the human breast cancer cohort. Correlation between vascular density (VE‐cadherin staining) and CD4 (D), vascular density and FOXP3 (E) and vascular density and CTLA4 (F) is also shown. (G) Correlation CD4 and PD‐L1 and (H) correlation CD163 and PD‐L1. A tumor with tumor cell CTLA4‐staining is also shown. (I) DAPI (blue), (J) CD4 (green) and FOXP3 (red), (K) FpA (gray) and VE‐cadherin (red) and (L) CTLA4 (yellow).
**Table S1.** IC cluster cell numbers.
**Table S2.** Extended list of signature markers in different myeloid clusters.
**Table S3.** Gene expression differences in myeloid, T cell and DC (dendritic cell) cell clusters.
**Table S4.** GO of gene expression changes listed in Table S3.
**Table S5.** EC (endothelial cell) gene expression changes.
**Table S6.** GO (gene ontology) categories of EC (endothelial cell) gene expresssion changes.
**Table S7.** Tumor and patient characteristics.
**Table S8.** List of immune reagents and software.

## Data Availability

EC bulk RNAseq data GEO accession number GSE293931: https://www.ncbi.nlm.nih.gov/geo/query/acc.cgi?acc=GSE293931. Single‐cell RNAseq of samples from Vav‐cre/Shb flox/flox mice GEO accession GSE294986: https://www.ncbi.nlm.nih.gov/geo/query/acc.cgi?acc=GSE294986. Single‐cell RNAseq of samples from Cdh5‐creERt2/Shb flox/flox mice GEO accession GSE294987: https://www.ncbi.nlm.nih.gov/geo/query/acc.cgi?acc=GSE294987. For additional information, please address the lead contact, Michael Welsh (michael.welsh@mcb.uu.se).

## References

[1] Hanahan D , Weinberg RA . Hallmarks of cancer: the next generation. Cell. 2011;144:646–674. 10.1016/j.cell.2011.02.013 21376230

[2] Schol P , van Elsas MJ , Middelburg J , Nijen Twilhaar MK , van Hall T , van der Sluis TC , et al. Myeloid effector cells in cancer. Cancer Cell. 2024;42:1997–2014. 10.1016/j.ccell.2024.11.002 39658540

[3] Cohen M , Giladi A , Barboy O , Hamon P , Li B , Zada M , et al. The interaction of CD4(+) helper T cells with dendritic cells shapes the tumor microenvironment and immune checkpoint blockade response. Nat Cancer. 2022;3:303–317. 10.1038/s43018-022-00338-5 35241835

[4] Geels SN , Moshensky A , Sousa RS , Murat C , Bustos MA , Walker BL , et al. Interruption of the intratumor CD8(+) T cell:Treg crosstalk improves the efficacy of PD‐1 immunotherapy. Cancer Cell. 2024;42:1051–1066.e1057. 10.1016/j.ccell.2024.05.013 38861924 PMC11285091

[5] Galassi C , Chan TA , Vitale I , Galluzzi L . The hallmarks of cancer immune evasion. Cancer Cell. 2024;42:1825–1863. 10.1016/j.ccell.2024.09.010 39393356

[6] Hegde S , Leader AM , Merad M . MDSC: markers, development, states, and unaddressed complexity. Immunity. 2021;54:875–884. 10.1016/j.immuni.2021.04.004 33979585 PMC8709560

[7] Franklin RA , Liao W , Sarkar A , Kim MV , Bivona MR , Liu K , et al. The cellular and molecular origin of tumor‐associated macrophages. Science. 2014;344:921–925. 10.1126/science.1252510 24812208 PMC4204732

[8] Mantovani A , Marchesi F , Malesci A , Laghi L , Allavena P . Tumour‐associated macrophages as treatment targets in oncology. Nat Rev Clin Oncol. 2017;14:399–416. 10.1038/nrclinonc.2016.217 28117416 PMC5480600

[9] Ferrara N . Vascular endothelial growth factor: basic science and clinical progress. Endocr Rev. 2004;25:581–611. 10.1210/er.2003-0027 15294883

[10] Claesson‐Welsh L . Vascular permeability—the essentials. Ups J Med Sci. 2015;120:135–143. 10.3109/03009734.2015.1064501 26220421 PMC4526869

[11] Claesson‐Welsh L , Dejana E , McDonald DM . Permeability of the endothelial barrier: identifying and reconciling controversies. Trends Mol Med. 2021;27:314–331. 10.1016/j.molmed.2020.11.006 33309601 PMC8005435

[12] Goel S , Wong AH , Jain RK . Vascular normalization as a therapeutic strategy for malignant and nonmalignant disease. Cold Spring Harb Perspect Med. 2012;2:a006486. 10.1101/cshperspect.a006486 22393532 PMC3282493

[13] Garcia‐Roman J , Zentella‐Dehesa A . Vascular permeability changes involved in tumor metastasis. Cancer Lett. 2013;335:259–269. 10.1016/j.canlet.2013.03.005 23499893

[14] Welsh M . Perspectives on vascular regulation of mechanisms controlling selective immune cell function in the tumor immune response. Int J Mol Sci. 2022;23:2313. 10.3390/ijms23042313 35216427 PMC8877013

[15] Dai P , Song T , Liu J , He Z , Wang X , Hu R , et al. Therapeutic strategies and landscape of metaplastic breast cancer. Cancer Treat Rev. 2025;133:102885. 10.1016/j.ctrv.2025.102885 39827533

[16] Cheruvu S , McMahon D , Larkin J . Navigating the landscape of immune checkpoint inhibitors and novel immunotherapies in melanoma: long‐term outcomes, progress, and challenges. Expert Opin Biol Ther. 2025;25:1–256. 10.1080/14712598.2025.2456485 39895540

[17] Sharma P , Hu‐Lieskovan S , Wargo JA , Ribas A . Primary, adaptive, and acquired resistance to cancer immunotherapy. Cell. 2017;168:707–723. 10.1016/j.cell.2017.01.017 28187290 PMC5391692

[18] Sun Q , Hong Z , Zhang C , Wang L , Han Z , Ma D . Immune checkpoint therapy for solid tumours: clinical dilemmas and future trends. Signal Transduct Target Ther. 2023;8:320. 10.1038/s41392-023-01522-4 37635168 PMC10460796

[19] Lugano R , Ramachandran M , Dimberg A . Tumor angiogenesis: causes, consequences, challenges and opportunities. Cell Mol Life Sci. 2020;77:1745–1770. 10.1007/s00018-019-03351-7 31690961 PMC7190605

[20] Jain RK . Normalizing tumor microenvironment to treat cancer: bench to bedside to biomarkers. J Clin Oncol. 2013;31:2205–2218. 10.1200/JCO.2012.46.3653 23669226 PMC3731977

[21] Nourshargh S , Alon R . Leukocyte migration into inflamed tissues. Immunity. 2014;41:694–707. 10.1016/j.immuni.2014.10.008 25517612

[22] Vestweber D . How leukocytes cross the vascular endothelium. Nat Rev Immunol. 2015;15:692–704. 10.1038/nri3908 26471775

[23] Facciabene A , Peng X , Hagemann IS , Balint K , Barchetti A , Wang LP , et al. Tumour hypoxia promotes tolerance and angiogenesis via CCL28 and T(reg) cells. Nature. 2011;475:226–230. 10.1038/nature10169 21753853

[24] Piao W , Wu L , Xiong Y , Zapas GC , Paluskievicz CM , Oakes RS , et al. Regulatory T cells crosstalk with tumor cells and endothelium through lymphotoxin signaling. Nat Commun. 2024;15:10468. 10.1038/s41467-024-54874-y 39622857 PMC11612289

[25] Piao W , Li L , Saxena V , Iyyathurai J , Lakhan R , Zhang Y , et al. PD‐L1 signaling selectively regulates T cell lymphatic transendothelial migration. Nat Commun. 2022;13:2176. 10.1038/s41467-022-29930-0 35449134 PMC9023578

[26] Akhtar S , Sagar K , Roy A , Hote MP , Arava S , Sharma A . CCR5‐mediated homing of regulatory T cells and monocytic‐myeloid derived suppressor cells to dysfunctional endothelium contributes to early atherosclerosis. Immunology. 2024;173:712–729. 10.1111/imm.13859 39256808

[27] Christoffersson G , Zang G , Zhuang ZW , Vagesjo E , Simons M , Phillipson M , et al. Vascular adaptation to a dysfunctional endothelium as a consequence of Shb deficiency. Angiogenesis. 2012;15:469–480. 10.1007/s10456-012-9275-z 22562363 PMC4059510

[28] Funa NS , Kriz V , Zang G , Calounova G , Akerblom B , Mares J , et al. Dysfunctional microvasculature as a consequence of shb gene inactivation causes impaired tumor growth. Cancer Res. 2009;69:2141–2148. 10.1158/0008-5472.CAN-08-3797 19223532

[29] He Q , Jamalpour M , Bergquist E , Anderson RL , Gustafsson K , Welsh M . Mouse breast carcinoma monocytic/macrophagic myeloid‐derived suppressor cell infiltration as a consequence of endothelial dysfunction in Shb‐deficient endothelial cells increases tumor lung metastasis. Int J Mol Sci. 2021;22:11478. 10.3390/ijms222111478 34768912 PMC8583852

[30] Vito A , Salem O , El‐Sayes N , MacFawn IP , Portillo AL , Milne K , et al. Immune checkpoint blockade in triple negative breast cancer influenced by B cells through myeloid‐derived suppressor cells. Commun Biol. 2021;4:859. 10.1038/s42003-021-02375-9 34253827 PMC8275624

[31] He Q , Li X , Singh K , Luo Z , Meija‐Cordova M , Jamalpour M , et al. The Cdh5‐CreERT2 transgene causes conditional Shb gene deletion in hematopoietic cells with consequences for immune cell responses to tumors. Sci Rep. 2019;9:7548. 10.1038/s41598-019-44039-z 31101877 PMC6525206

[32] Ramilowski JA , Goldberg T , Harshbarger J , Kloppmann E , Lizio M , Satagopam VP , et al. A draft network of ligand‐receptor‐mediated multicellular signalling in human. Nat Commun. 2015;6:7866. 10.1038/ncomms8866 26198319 PMC4525178

[33] Collin M , Bigley V . Human dendritic cell subsets: an update. Immunology. 2018;154:3–20. 10.1111/imm.12888 29313948 PMC5904714

[34] Guimaraes GR , Maklouf GR , Teixeira CE , de Oliveira SL , Tessarollo NG , de Toledo NE , et al. Single‐cell resolution characterization of myeloid‐derived cell states with implication in cancer outcome. Nat Commun. 2024;15:5694. 10.1038/s41467-024-49916-4 38972873 PMC11228020

[35] Hameed A , Hruban RH , Gage W , Pettis G , Fox WM 3rd . Immunohistochemical expression of CD68 antigen in human peripheral blood T cells. Hum Pathol. 1994;25:872–876. 10.1016/0046-8177(94)90005-1 8088761

[36] Sasaki M , Miyakoshi M , Sato Y , Nakanuma Y . Chemokine‐chemokine receptor CCL2‐CCR2 and CX3CL1‐CX3CR1 axis may play a role in the aggravated inflammation in primary biliary cirrhosis. Dig Dis Sci. 2014;59:358–364. 10.1007/s10620-013-2920-6 24185682

[37] Mahadevan KK , Dyevoich AM , Chen Y , Li B , Sugimoto H , Sockwell AM , et al. Type I conventional dendritic cells facilitate immunotherapy in pancreatic cancer. Science. 2024;384:eadh4567. 10.1126/science.adh4567 38935717 PMC11841451

[38] Waibl Polania J , Hoyt‐Miggelbrink A , Tomaszewski WH , Wachsmuth LP , Lorrey SJ , Wilkinson DS , et al. Antigen presentation by tumor‐associated macrophages drives T cells from a progenitor exhaustion state to terminal exhaustion. Immunity. 2025;58:232–246.e236. 10.1016/j.immuni.2024.11.026 39724910

[39] Rosenlehner T , Pennavaria S , Akcabozan B , Jahani S , O'Neill TJ , Krappmann D , et al. Reciprocal regulation of mTORC1 signaling and ribosomal biosynthesis determines cell cycle progression in activated T cells. Sci Signal. 2024;17:eadi8753. 10.1126/scisignal.adi8753 39436996

[40] Pinjusic K , Dubey OA , Egorova O , Nassiri S , Meylan E , Faget J , et al. Activin‐a impairs CD8 T cell‐mediated immunity and immune checkpoint therapy response in melanoma. J Immunother Cancer. 2022;10:e004533. 10.1136/jitc-2022-004533 35580932 PMC9125758

[41] Li S , Liu M , Do MH , Chou C , Stamatiades EG , Nixon BG , et al. Cancer immunotherapy via targeted TGF‐beta signalling blockade in T(H) cells. Nature. 2020;587:121–125. 10.1038/s41586-020-2850-3 33087933 PMC8353603

[42] McGinnis CS , Miao Z , Superville D , Yao W , Goga A , Reticker‐Flynn NE , et al. The temporal progression of lung immune remodeling during breast cancer metastasis. Cancer Cell. 2024;42:1018–1031.e1016. 10.1016/j.ccell.2024.05.004 38821060 PMC11255555

[43] Korbecki J , Grochans S , Gutowska I , Barczak K , Baranowska‐Bosiacka I . CC chemokines in a tumor: a review of pro‐cancer and anti‐cancer properties of receptors CCR5, CCR6, CCR7, CCR8, CCR9, and CCR10 ligands. Int J Mol Sci. 2020;21:7619. 10.3390/ijms21207619 33076281 PMC7590012

[44] Korbecki J , Kojder K , Siminska D , Bohatyrewicz R , Gutowska I , Chlubek D , et al. CC chemokines in a tumor: a review of pro‐cancer and anti‐cancer properties of the ligands of receptors CCR1, CCR2, CCR3, and CCR4. Int J Mol Sci. 2020;21:8412. 10.3390/ijms21218412 33182504 PMC7665155

[45] Li BH , Garstka MA , Li ZF . Chemokines and their receptors promoting the recruitment of myeloid‐derived suppressor cells into the tumor. Mol Immunol. 2020;117:201–215. 10.1016/j.molimm.2019.11.014 31835202

[46] Li H , Wu M , Zhao X . Role of chemokine systems in cancer and inflammatory diseases. MedComm. 2022;3:e147. 10.1002/mco2.147 35702353 PMC9175564

[47] You S , Li S , Zeng L , Song J , Li Z , Li W , et al. Lymphatic‐localized Treg‐mregDC crosstalk limits antigen trafficking and restrains anti‐tumor immunity. Cancer Cell. 2024;42:1415–1433.e1412. 10.1016/j.ccell.2024.06.014 39029466

[48] Hanggi K , Li J , Gangadharan A , Liu X , Celias DP , Osunmakinde O , et al. Interleukin‐1alpha release during necrotic‐like cell death generates myeloid‐driven immunosuppression that restricts anti‐tumor immunity. Cancer Cell. 2024;42:2015–2031.e2011. 10.1016/j.ccell.2024.10.014 39577420 PMC11631672

[49] Susek KH , Karvouni M , Alici E , Lundqvist A . The role of CXC chemokine receptors 1‐4 on immune cells in the tumor microenvironment. Front Immunol. 2018;9:2159. 10.3389/fimmu.2018.02159 30319622 PMC6167945

[50] Moreno Ayala MA , Campbell TF , Zhang C , Dahan N , Bockman A , Prakash V , et al. CXCR3 expression in regulatory T cells drives interactions with type I dendritic cells in tumors to restrict CD8(+) T cell antitumor immunity. Immunity. 2023;56:1613–1630.e1615. 10.1016/j.immuni.2023.06.003 37392735 PMC10752240

[51] Huang C , Wang X , Wang Y , Feng Y , Wang X , Chen S , et al. Sirpalpha on tumor‐associated myeloid cells restrains antitumor immunity in colorectal cancer independent of its interaction with CD47. Nat Cancer. 2024;5:500–516. 10.1038/s43018-023-00691-z 38200243

[52] Pence BD . Growth differentiation Factor‐15 in immunity and aging. Front Aging. 2022;3:837575. 10.3389/fragi.2022.837575 35821815 PMC9261309

[53] Sweet DR , Lam C , Jain MK . Evolutionary protection of Kruppel‐like factors 2 and 4 in the development of the mature Hemovascular system. Front Cardiovasc Med. 2021;8:645719. 10.3389/fcvm.2021.645719 34079826 PMC8165158

[54] Ren FJ , Cai XY , Yao Y , Fang GY . JunB: a paradigm for Jun family in immune response and cancer. Front Cell Infect Microbiol. 2023;13:1222265. 10.3389/fcimb.2023.1222265 37731821 PMC10507257

[55] Haensel D , Daniel B , Gaddam S , Pan C , Fabo T , Bjelajac J , et al. Skin basal cell carcinomas assemble a pro‐tumorigenic spatially organized and self‐propagating Trem2+ myeloid niche. Nat Commun. 2023;14:2685. 10.1038/s41467-023-37993-w 37164949 PMC10172319

[56] Puttock EH , Tyler EJ , Manni M , Maniati E , Butterworth C , Burger Ramos M , et al. Extracellular matrix educates an immunoregulatory tumor macrophage phenotype found in ovarian cancer metastasis. Nat Commun. 2023;14:2514. 10.1038/s41467-023-38093-5 37188691 PMC10185550

[57] Tang HC , Lai YY , Zheng J , Jiang HY , Xu G . miR‐223‐3p inhibits antigen endocytosis and presentation and promotes the tolerogenic potential of dendritic cells through targeting mannose receptor signaling and Rhob. J Immunol Res. 2020;2020:1379458. 10.1155/2020/1379458 32656268 PMC7320286

[58] Xue Y , Yan X , Li D , Dong S , Ping Y . Proinflammatory polarization of engineered heat‐inducible macrophages reprogram the tumor immune microenvironment during cancer immunotherapy. Nat Commun. 2024;15:2270. 10.1038/s41467-024-46210-1 38491004 PMC10943244

[59] Zhong J , Xing X , Gao Y , Pei L , Lu C , Sun H , et al. Distinct roles of TREM2 in central nervous system cancers and peripheral cancers. Cancer Cell. 2024;42:968–984.e969. 10.1016/j.ccell.2024.05.001 38788719

[60] Zheng M , Zhang Z , Yu L , Wang Z , Dong Y , Tong A , et al. Immune‐checkpoint protein VISTA in allergic, autoimmune disease and transplant rejection. Front Immunol. 2023;14:1194421. 10.3389/fimmu.2023.1194421 37435070 PMC10330820

[61] Bejarano L , Kauzlaric A , Lamprou E , Lourenco J , Fournier N , Ballabio M , et al. Interrogation of endothelial and mural cells in brain metastasis reveals key immune‐regulatory mechanisms. Cancer Cell. 2024;42:378–395.e310. 10.1016/j.ccell.2023.12.018 38242126

[62] Mortezaee K . B7‐H3 immunoregulatory roles in cancer. Biomed Pharmacother. 2023;163:114890. 10.1016/j.biopha.2023.114890 37196544

[63] Liu CW , Wu LS , Lin CJ , Wu HC , Liu KC , Lee SW . Association of tuberculosis risk with genetic polymorphisms of the immune checkpoint genes PDCD1, CTLA‐4, and TIM3. PLoS One. 2024;19:e0303431. 10.1371/journal.pone.0303431 38723011 PMC11081348

[64] Guan X , Hu R , Choi Y , Srivats S , Nabet BY , Silva J , et al. Anti‐TIGIT antibody improves PD‐L1 blockade through myeloid and T(reg) cells. Nature. 2024;627:646–655. 10.1038/s41586-024-07121-9 38418879 PMC11139643

[65] Jiang VC , Hao D , Jain P , Li Y , Cai Q , Yao Y , et al. TIGIT is the central player in T‐cell suppression associated with CAR T‐cell relapse in mantle cell lymphoma. Mol Cancer. 2022;21:185. 10.1186/s12943-022-01655-0 36163179 PMC9513944

[66] He J , Xiong X , Yang H , Li D , Liu X , Li S , et al. Defined tumor antigen‐specific T cells potentiate personalized TCR‐T cell therapy and prediction of immunotherapy response. Cell Res. 2022;32:530–542. 10.1038/s41422-022-00627-9 35165422 PMC9160085

[67] Wang X , Zha H , Wu W , Yuan T , Xie S , Jin Z , et al. CD200(+) cytotoxic T lymphocytes in the tumor microenvironment are crucial for efficacious anti‐PD‐1/PD‐L1 therapy. Sci Transl Med. 2023;15:eabn5029. 10.1126/scitranslmed.abn5029 36652534

[68] Hossen MM , Ma Y , Yin Z , Xia Y , Du J , Huang JY , et al. Current understanding of CTLA‐4: from mechanism to autoimmune diseases. Front Immunol. 2023;14:1198365. 10.3389/fimmu.2023.1198365 37497212 PMC10367421

[69] Wang S , Wang B , Shi Y , Moller T , Stegmeyer RI , Strilic B , et al. Mechanosensation by endothelial PIEZO1 is required for leukocyte diapedesis. Blood. 2022;140:171–183. 10.1182/blood.2021014614 35443048 PMC9305087

[70] Farber MJ , Rizaldy R , Hildebrand JD . Shroom2 regulates contractility to control endothelial morphogenesis. Mol Biol Cell. 2011;22:795–805. 10.1091/mbc.E10-06-0505 21248203 PMC3057704

[71] Lobov I , Mikhailova N . The role of Dll4/notch signaling in Normal and pathological ocular angiogenesis: Dll4 controls blood vessel sprouting and vessel remodeling in Normal and pathological conditions. J Ophthalmol. 2018;2018:3565292. 10.1155/2018/3565292 30116629 PMC6079472

[72] Torres‐Torres J , Espino YSS , Martinez‐Portilla R , Borboa‐Olivares H , Estrada‐Gutierrez G , Acevedo‐Gallegos S , et al. A narrative review on the pathophysiology of preeclampsia. Int J Mol Sci. 2024;25:7569. 10.3390/ijms25147569 39062815 PMC11277207

[73] Sjoberg E , Melssen M , Richards M , Ding Y , Chanoca C , Chen D , et al. Endothelial VEGFR2‐PLCγ signaling regulates vascular permeability and antitumor immunity through eNOS/Src. J Clin Invest. 2023;133(20):e161366. 10.1172/JCI161366 37651195 PMC10575733

[74] Baeyens N , Mulligan‐Kehoe MJ , Corti F , Simon DD , Ross TD , Rhodes JM , et al. Syndecan 4 is required for endothelial alignment in flow and atheroprotective signaling. Proc Natl Acad Sci U S A. 2014;111:17308–17313. 10.1073/pnas.1413725111 25404299 PMC4260558

[75] De Rossi G , Vahatupa M , Cristante E , Arokiasamy S , Liyanage SE , May U , et al. Pathological angiogenesis requires Syndecan‐4 for efficient VEGFA‐induced VE‐cadherin internalization. Arterioscler Thromb Vasc Biol. 2021;41:1374–1389. 10.1161/ATVBAHA.121.315941 33596666 PMC7613699

[76] Fears CY , Gladson CL , Woods A . Syndecan‐2 is expressed in the microvasculature of gliomas and regulates angiogenic processes in microvascular endothelial cells. J Biol Chem. 2006;281:14533–14536. 10.1074/jbc.C600075200 16574663

[77] Fournier P , Dussault S , Fusco A , Rivard A , Royal I . Tyrosine phosphatase PTPRJ/DEP‐1 is an essential promoter of vascular permeability, angiogenesis, and tumor progression. Cancer Res. 2016;76:5080–5091. 10.1158/0008-5472.CAN-16-1071 27364551

[78] Jin Y , Ding Y , Richards M , Kaakinen M , Giese W , Baumann E , et al. Tyrosine‐protein kinase yes controls endothelial junctional plasticity and barrier integrity by regulating VE‐cadherin phosphorylation and endocytosis. Nat Cardiovasc Res. 2022;1:1156–1173. 10.1038/s44161-022-00172-z 37936984 PMC7615285

[79] Lin JB , Sene A , Wiley LA , Santeford A , Nudleman E , Nakamura R , et al. WNT7A/B promote choroidal neovascularization. Exp Eye Res. 2018;174:107–112. 10.1016/j.exer.2018.05.033 29864439 PMC6110966

[80] Mezzapelle R , Leo M , Caprioglio F , Colley LS , Lamarca A , Sabatino L , et al. CXCR4/CXCL12 activities in the tumor microenvironment and implications for tumor immunotherapy. Cancers (Basel). 2022;14:2314. 10.3390/cancers14092314 35565443 PMC9105267

[81] Rapraeger AC , Ell BJ , Roy M , Li X , Morrison OR , Thomas GM , et al. Vascular endothelial‐cadherin stimulates syndecan‐1‐coupled insulin‐like growth factor‐1 receptor and cross‐talk between alphaVbeta3 integrin and vascular endothelial growth factor receptor 2 at the onset of endothelial cell dissemination during angiogenesis. FEBS J. 2013;280:2194–2206. 10.1111/febs.12134 23331867 PMC3640762

[82] Spring K , Chabot C , Langlois S , Lapointe L , Trinh NT , Caron C , et al. Tyrosine phosphorylation of DEP‐1/CD148 as a mechanism controlling Src kinase activation, endothelial cell permeability, invasion, and capillary formation. Blood. 2012;120:2745–2756. 10.1182/blood-2011-12-398040 22898603

[83] Groppa E , Brkic S , Bovo E , Reginato S , Sacchi V , Di Maggio N , et al. VEGF dose regulates vascular stabilization through Semaphorin3A and the Neuropilin‐1 + monocyte/TGF‐β1 paracrine axis. EMBO Mol Med. 2015;7(10):1366–1384. 10.15252/emmm.201405003 26323572 PMC4604689

[84] Fearnley GW , Young KA , Edgar JR , Antrobus R , Hay IM , Liang WC , et al. The homophilic receptor PTPRK selectively dephosphorylates multiple junctional regulators to promote cell‐cell adhesion. Elife. 2019;8:e44597. 10.7554/eLife.44597 30924770 PMC6440744

[85] Uutela M , Wirzenius M , Paavonen K , Rajantie I , He Y , Karpanen T , et al. PDGF‐D induces macrophage recruitment, increased interstitial pressure, and blood vessel maturation during angiogenesis. Blood. 2004;104:3198–3204. 10.1182/blood-2004-04-1485 15271796

[86] Vestweber D . Vascular endothelial protein tyrosine phosphatase regulates endothelial function. Physiology (Bethesda). 2021;36:84–93. 10.1152/physiol.00026.2020 33595386

[87] Demolli S , Doddaballapur A , Devraj K , Stark K , Manavski Y , Eckart A , et al. Shear stress‐regulated miR‐27b controls pericyte recruitment by repressing SEMA6A and SEMA6D. Cardiovasc Res. 2017;113:681–691. 10.1093/cvr/cvx032 28453731

[88] Liu H , Devraj K , Moller K , Liebner S , Hecker M , Korff T . EphrinB‐mediated reverse signalling controls junctional integrity and pro‐inflammatory differentiation of endothelial cells. Thromb Haemost. 2014;112:151–163. 10.1160/TH13-12-1034 24522257

[89] Zhou YF , Li YN , Jin HJ , Wu JH , He QW , Wang XX , et al. Sema4D/PlexinB1 inhibition ameliorates blood‐brain barrier damage and improves outcome after stroke in rats. FASEB J. 2018;32:2181–2196. 10.1096/fj.201700786RR 29242274

[90] Fu T , Sullivan DP , Gonzalez AM , Haynes ME , Dalal PJ , Rutledge NS , et al. Mechanotransduction via endothelial adhesion molecule CD31 initiates transmigration and reveals a role for VEGFR2 in diapedesis. Immunity. 2023;56:2311–2324.e2316. 10.1016/j.immuni.2023.08.001 37643615 PMC11670454

[91] Liu D , Zhong M , Zhan D , Zhang Y , Liu S . Roles of the HGF/met signaling in head and neck squamous cell carcinoma: focus on tumor immunity (review). Oncol Rep. 2020;44:2337–2344. 10.3892/or.2020.7799 33125120

[92] Huang F , Hu L , Zhang Y , Qu X , Xu J . BMP4 moderates glycolysis and regulates activation and interferon‐gamma production in CD4+ T cells. Front Immunol. 2021;12:702211. 10.3389/fimmu.2021.702211 34413854 PMC8368433

[93] Arisz RA , de Vries JJ , Schols SEM , Eikenboom JCJ , de Maat MPM . Interaction of von Willebrand factor with blood cells in flow models: a systematic review. Blood Adv. 2022;6:3979–3990. 10.1182/bloodadvances.2021006405 35816358 PMC9278308

[94] Hossain F , Majumder S , Ucar DA , Rodriguez PC , Golde TE , Minter LM , et al. Notch signaling in myeloid cells as a regulator of tumor immune responses. Front Immunol. 2018;9:1288. 10.3389/fimmu.2018.01288 29915603 PMC5994797

[95] Keewan E , Naser SA . The role of notch signaling in macrophages during inflammation and infection: implication in rheumatoid arthritis? Cells. 2020;9:111. 10.3390/cells9010111 31906482 PMC7016800

[96] Manes TD , Wang V , Pober JS . Costimulators expressed on human endothelial cells modulate antigen‐dependent recruitment of circulating T lymphocytes. Front Immunol. 2022;13:1016361. 10.3389/fimmu.2022.1016361 36275645 PMC9582530

[97] Solinas C , Gu‐Trantien C , Willard‐Gallo K . The rationale behind targeting the ICOS‐ICOS ligand costimulatory pathway in cancer immunotherapy. ESMO Open. 2020;5:e000544. 10.1136/esmoopen-2019-000544 32516116 PMC7003380

[98] Larimore K , Liang L , Bakkour S , Sha WC . B7h‐expressing dendritic cells and plasma B cells mediate distinct outcomes of ICOS costimulation in T cell‐dependent antibody responses. BMC Immunol. 2012;13:29. 10.1186/1471-2172-13-29 22686515 PMC3477010

[99] Aneesh Kumar A , Ajith Kumar GS , Satheesh G , Surendran A , Chandran M , Kartha CC , et al. Proteomics analysis reveals diverse molecular characteristics between endocardial and aortic‐valvular endothelium. Genes (Basel). 2021;12:1005. 10.3390/genes12071005 34208790 PMC8304717

[100] Hueso L , Marques P , Morant B , Gonzalez‐Navarro H , Ortega J , Real JT , et al. CCL17 and CCL22 chemokines are upregulated in human obesity and play a role in vascular dysfunction. Front Endocrinol (Lausanne). 2023;14:1154158. 10.3389/fendo.2023.1154158 37124725 PMC10130371

[101] Piao W , Xiong Y , Li L , Saxena V , Smith KD , Hippen KL , et al. Regulatory T cells condition lymphatic endothelia for enhanced Transendothelial migration. Cell Rep. 2020;30:1052–1062.e1055. 10.1016/j.celrep.2019.12.083 31995749 PMC7009789

[102] Yeung L , Hickey MJ , Wright MD . The many and varied roles of Tetraspanins in immune cell recruitment and migration. Front Immunol. 2018;9:1644. 10.3389/fimmu.2018.01644 30072994 PMC6060431

[103] Gemmell E , Carter CL , Hart DN , Drysdale KE , Seymour GJ . Antigen‐presenting cells in human periodontal disease tissues. Oral Microbiol Immunol. 2002;17:388–393. 10.1034/j.1399-302x.2002.170609.x 12485331

[104] Katsuta E , Maawy AA , Yan L , Takabe K . High expression of bone morphogenetic protein (BMP) 6 and BMP7 are associated with higher immune cell infiltration and better survival in estrogen receptor‐positive breast cancer. Oncol Rep. 2019;42:1413–1421. 10.3892/or.2019.7275 31524275 PMC6718975

[105] Tjwa M , Bellido‐Martin L , Lin Y , Lutgens E , Plaisance S , Bono F , et al. Gas6 promotes inflammation by enhancing interactions between endothelial cells, platelets, and leukocytes. Blood. 2008;111:4096–4105. 10.1182/blood-2007-05-089565 18156494

[106] Vockel M , Vestweber D . How T cells trigger the dissociation of the endothelial receptor phosphatase VE‐PTP from VE‐cadherin. Blood. 2013;122:2512–2522. 10.1182/blood-2013-04-499228 23908467

[107] Pober JS , Merola J , Liu R , Manes TD . Antigen presentation by vascular cells. Front Immunol. 2017;8:1907. 10.3389/fimmu.2017.01907 29312357 PMC5744398

[108] Snelgrove SL , Abeynaike LD , Thevalingam S , Deane JA , Hickey MJ . Regulatory T cell transmigration and intravascular migration undergo mechanistically distinct regulation at different phases of the inflammatory response. J Immunol. 2019;203:2850–2861. 10.4049/jimmunol.1900447 31653684

[109] Walchli T , Ghobrial M , Schwab M , Takada S , Zhong H , Suntharalingham S , et al. Single‐cell atlas of the human brain vasculature across development, adulthood and disease. Nature. 2024;632:603–613. 10.1038/s41586-024-07493-y 38987604 PMC11324530

[110] Zhu H , Yin L , Sun Y , Xu Y , Ma J , Qiao L . Identification and validation of signature genes and their correlations with infiltrated immune cells in neonatal sepsis. Altern Ther Health Med. 2023;29:272–277. 37471663

[111] Lu L , Li J , Jiang X , Bai R . CXCR4/CXCL12 axis: “old” pathway as “novel” target for anti‐inflammatory drug discovery. Med Res Rev. 2024;44:1189–1220. 10.1002/med.22011 38178560

[112] Wu L , Islam MR , Lee J , Takase H , Guo S , Andrews AM , et al. ErbB3 is a critical regulator of cytoskeletal dynamics in brain microvascular endothelial cells: implications for vascular remodeling and blood brain barrier modulation. J Cereb Blood Flow Metab. 2021;41:2242–2255. 10.1177/0271678X20984976 33583260 PMC8393293

[113] Melderis S , Hagenstein J , Warkotsch MT , Dang J , Herrnstadt GR , Niehus CB , et al. Amphiregulin aggravates glomerulonephritis via recruitment and activation of myeloid cells. J Am Soc Nephrol. 2020;31:1996–2012. 10.1681/ASN.2019111215 32616537 PMC7461677

[114] Zhou D , Liu L , Liu J , Li H , Zhang J , Cao Z . A systematic review of the advances in the study of T lymphocyte suppressor receptors in HBV infection: potential therapeutic targets. J Clin Med. 2024;13:1210. 10.3390/jcm13051210 38592036 PMC10931645

[115] Barbarulo A , Lau CI , Mengrelis K , Ross S , Solanki A , Saldana JI , et al. Hedgehog Signalling in the embryonic mouse thymus. J Dev Biol. 2016;4:22. 10.3390/jdb4030022 27504268 PMC4975939

[116] Zhong Y , Zhang X , Chong W . Interleukin‐24 immunobiology and its roles in inflammatory diseases. Int J Mol Sci. 2022;23:627. 10.3390/ijms23020627 35054813 PMC8776082

[117] Terracina S , Ferraguti G , Tarani L , Fanfarillo F , Tirassa P , Ralli M , et al. Nerve growth factor and autoimmune diseases. Curr Issues Mol Biol. 2023;45:8950–8973. 10.3390/cimb45110562 37998739 PMC10670231

[118] Komai T , Okamura T , Inoue M , Yamamoto K , Fujio K . Reevaluation of pluripotent cytokine TGF‐beta3 in immunity. Int J Mol Sci. 2018;19:2261. 10.3390/ijms19082261 30071700 PMC6121403

[119] Kawecki C , Lenting PJ , Denis CV . von Willebrand factor and inflammation. J Thromb Haemost. 2017;15:1285–1294. 10.1111/jth.13696 28671350

[120] Zhou C , Sun T , Dong Z , Lu F , Li B . The interplay between lymphatic vessels and macrophages in inflammation response. FASEB J. 2024;38:e23879. 10.1096/fj.202400160RR 39162663

[121] Lennard AC . lnterleukin‐1 receptor antagonist. Crit Rev Immunol. 2017;37:531–559. 10.1615/CritRevImmunol.v37.i2-6.160 29773033

[122] Nwadozi E , Strell C , Nordling S , Backman M , Marwitz S , Goldmann T , et al. Vascular leakage in human non‐small cell lung cancer predicts immune evasion and poor response to immunotherapy. 2024. Preprint. 10.2139/ssrn.4937462 42486347

[123] Kowal K , Silver R , Slawinska E , Bielecki M , Chyczewski L , Kowal‐Bielecka O . CD163 and its role in inflammation. Folia Histochem Cytobiol. 2011;49:365–374. 10.5603/fhc.2011.0052 22038213

[124] Svendsen P , Etzerodt A , Deleuran BW , Moestrup SK . Mouse CD163 deficiency strongly enhances experimental collagen‐induced arthritis. Sci Rep. 2020;10:12447. 10.1038/s41598-020-69018-7 32710083 PMC7382459

[125] Guo X , Nie H , Zhang W , Li J , Ge J , Xie B , et al. Contrasting cytotoxic and regulatory T cell responses underlying distinct clinical outcomes to anti‐PD‐1 plus lenvatinib therapy in cancer. Cancer Cell. 2025;43:248–268.e249. 10.1016/j.ccell.2025.01.001 39889705

[126] Fukumura D , Kloepper J , Amoozgar Z , Duda DG , Jain RK . Enhancing cancer immunotherapy using antiangiogenics: opportunities and challenges. Nat Rev Clin Oncol. 2018;15:325–340. 10.1038/nrclinonc.2018.29 29508855 PMC5921900

[127] Qian C , Liu C , Liu W , Zhou R , Zhao L . Targeting vascular normalization: a promising strategy to improve immune‐vascular crosstalk in cancer immunotherapy. Front Immunol. 2023;14:1291530. 10.3389/fimmu.2023.1291530 38193080 PMC10773740

[128] Zhulai G , Oleinik E . Targeting regulatory T cells in anti‐PD‐1/PD‐L1 cancer immunotherapy. Scand J Immunol. 2022;95:e13129. 10.1111/sji.13129 34936125

