# Supplementary material for: Reduced vascular leakage correlates with breast carcinoma T regulatory cell infiltration but not with metastatic propensity
Source: Mol Oncol. 2025 Oct 16;20(3):753–78. doi: 10.1002/1878-0261.70144 (PMC13042803; doi:10.1002/1878-0261.70144)
Supplement: Supplementary file 1 — Fig. S1. Cluster expression of signature macrophage and monocyte gene markers. Fig. S2. Cluster expression of signature T cell and DC (dendritic cell) gene markers. Fig. S3. (A) Typical staining for VE‐cadherin (vascular, red), FpA (leakage, gray), and CD4+‐cells (green). (B) DAPI of the same section. Tumor boundary has been indicated. (C) CD8 (white) and GZMB (green) or (D) PD1 (red). (E, F) CD163 (yellow) and PD‐L1 (red) in human breast cancer stroma. Fig. S4. Correlations between CD4 and FOXP3 (A), CTLA4 and CD4 (B) and CTLA4 and FOXP3/CD4 (C) in the human breast cancer cohort. Correlation between vascular density (VE‐cadherin staining) and CD4 (D), vascular density and FOXP3 (E) and vascular density and CTLA4 (F) is also shown. (G) Correlation CD4 and PD‐L1 and (H) correlation CD163 and PD‐L1. A tumor with tumor cell CTLA4‐staining is also shown. (I) DAPI (blue), (J) CD4 (green) and FOXP3 (red), (K) FpA (gray) and VE‐cadherin (red) and (L) CTLA4 (yellow). Table S1. IC cluster cell numbers. Table S2. Extended list of signature markers in different myeloid clusters. Table S3. Gene expression differences in myeloid, T cell and DC (dendritic cell) cell clusters. Table S4. GO of gene expression changes listed in Table S3. Table S5. EC (endothelial cell) gene expression changes. Table S6. GO (gene ontology) categories of EC (endothelial cell) gene expresssion changes. Table S7. Tumor and patient characteristics. Table S8. List of immune reagents and software. [file MOL2-20-753-s001.zip › mol270144-sup-0001-supinfo.docx]

**Legends to supplementary figures**

Figure S1: Cluster expression of signature macrophage and monocyte gene markers.

Figure S2: Cluster expression of signature T cell and DC (dendritic cell) gene markers. In addition, UMAP (uniform manifold approximation and projection) plots of Cd68/Cd8a, Cd68/Cd4, Cd68/Cd3e and Cd4/Foxp3 double positive cells are shown in clusters 7, 12, 19 and 26.

Figure S3: (A) Typical staining for VE-cadherin (vascular, red), FpA (leakage, grey) and CD4^+^-cells (green). (B) DAPI of the same section. Tumor boundary has been indicated. (C) CD8 (white) and GZMB (green) or (D) PD1 (red). (E, F) CD163 (yellow) and PD-L1 (red) in human breast cancer stroma. Staining was performed 20 times on sections from 20 patients. Scale bars of 100 μm are shown, for A, B below, outside images, for C-F inside images. PD-L1= programmed death ligand 1, GZMB= granzyme B, PD= programmed death, VE= vascular endotherlial.

Figure S4: Correlations between CD4 and FOXP3 (A), CTLA4 and CD4 (B) and CTLA4 and FOXP3/CD4 (C) in the human breast cancer cohort. Correlation between vascular density (VE-cadherin staining) and CD4 (D), vascular density and FOXP3 (E) and vascular density and CTLA4 (F) is also shown. (G) Correlation CD4 and PD-L1 and (H) correlation CD163 and PD-L1. A tumor with tumor cell CTLA4-staining is also shown. (I) DAPI (blue), (J) CD4 (green) and FOXP3 (red), (K) FpA (grey) and VE-cadherin (red) and (L) CTLA4 (yellow). The boxed area in J has been expanded. Correlation coefficients (r) in A-H are 0.954, 0.800, 0.628, 0.638, 0.616, 0.500, 0.737 and 0.609, respectively. FpA: fibrinogen fragment indicative of leakage, Treg= T regulatory cells, CTLA4= cytotoxic T lymphocyte associated protein 4, PD-L1= programmed death ligand 1, FOXP3= transcription factor indicative of Treg, VE-vascular endothelial. Scale bar 250 μm for i-l and scale bar 150 μm for j inset have been indicated. A-L are based on 20 separate stains from 20 separate patients. P values for Pearson r correlations values are given in the graphs.

**Supporting tables S1, S2, S7, S8 and legends to supporting tables S3-6**

Table S1: IC cluster cell numbers.

| Cluster | Wild type (percent of CD45+ cells) | *Shb* iECKO (percent of CD45+ cells) |
| --- | --- | --- |
| 0 macrophages | 9.2 ± 1.0 | 12.7 ± 1.5 (p=0.09) |
| 1 macrophages | 9.1 ± 3.1 | 7.1 ± 1.0 |
| 2 monocytes/macrophages | 9.0 ± 1.0 | 10.3 ± 0.8 |
| 3 macrophages | 7.1 ± 0.8 | 8.2 ± 1.5 |
| 4 macrophages | 5.2 ± 0.6 | 7.8 ± 0.5 ** |
| 5 macrophages | 5.6 ± 0.7 | 4.6 ± 0.9 |
| 6 macrophages | 7.1 ± 0.9 | 5.7 ± 1.0 |
| 7 Cd68+ T cells | 2.9 ± 1.2 | 3.3 ± 1.6 |
| 8 macrophages | 6.6 ± 1.4 | 4.1 ± 0.6 |
| 9 cDC2 | 3.7 ± 0.2 | 3.7 ± 0.3 |
| 10 macrophages | 3.5 ± 0.6 | 4.6 ± 1.0 |
| 11 mregDC, cDC2 | 2.8 ± 0.2 | 2.9 ± 0.3 |
| 12 T cells | 4.6 ± 1.3 | 4.7 ± 1.1 |
| 13 monocytes/macrophages | 1.3 ± 0.7 | 1.7 ± 1.0 |
| 15 macrophages | 2.4 ± 0.2 | 2.3 ± 0.4 |
| 17 macrophages | 2.7 ± 0.3 | 1.5 ± 0.2 * |
| 18 macrophages | 2.8 ± 0.5 | 1.9 ± 0.3 |
| 19 Cd68+ T cells, Tregs | 1.4 ± 0.4 | 1.3 ± 0.3 |
| 21 pDC | 1.1 ± 0.3 | 1.0 ± 0.1 |
| 24 neutrophils | 1.2 ± 0.3 | 1.4 ± 0.4 |
| 25 cDC1 | 1.1 ± 0.1 | 0.8 ± 0.1 ** |
| 26 Tregs | 1.5 ± 0.6 | 1.6 ± 0.6 |
| 27 B cells | 0.7 ± 0.2 | 0.3 ± 0.1 |
| 29 NK cells | 0.6 ± 0.2 | 0.5 ± 0.2 |

Percentages cluster cell number ± SEM for the 6 individal tumors in three separate experiments. * indicates p<0.05 and ** p<0.01. respectively, by Students’ t-test. IC= immune cells, Treg= T regulatory cell.

Table S2: Extended list of signature markers in different myeloid clusters.

| cluster | Gene signature |
| --- | --- |
| 0 | *Lyz2, Cd68, Itgam, Ccr2, Il1b, Cd14, Csf1r, Cxcl9, Ly6a, Ly6i, Ms4a4c, Pdcd1lg2* (PD-L2)*, Tgfb1* |
| 1 | *Cd163, Ms4a4a* |
| 2 | *Lyz2, Ccr2, Il1b, Cd14, Sell, Ly6c2, Csf1r, Vcan, Tgfb1* |
| 3 | *Lyz2, Cd68, Itgam, Adgre1* (F4/80)*, Mrc1, Ccl8, Csf1r, Apoe, Gatm, Ly6a, Cxcl2, Pdcd1lg2* (PD-L2)*, Trem2, Havcr2, Tgfb1, Cxcl9* |
| 4 | *Lyz2, Cd68, Itgam, Ccr2, Il1b, Sell, Ly6c2, Csf1r, Vcan, Cxcl9, Ly6a, Ly6i, Ms4a4c, Cd274* (PD-L1) |
| 5 | *Lyz2, Cd68, Itgam, Adgre1* (F4/80)*, Mrc1, Csf1r, Apoe, Gatm, Trem2, Mki67* |
| 6 | *Lyz2, Ccr2, Mrc1, Cd14, Csf1r, Gatm, Cxcl2, Inhba, Pdcd1lg2* (PD-L2)*, Trem2, Tgfb1, Lpl, Lipa, Mgl2* |
| 8 | *Lyz2, Cd68, Cd163, Itgam, Arg1, Mrc1, Ccl8, Cd14, Csf1r, Apoe, Cxcl2, Pdcd1lg2* (PD-L2)*, Trem2, Folr2, Tgfb1, Adgre1* (F4/80), *Ms4a4a, Lyve1, Cxcl1, Tgfb1* |
| 10 | *Lyz2, Cd68, Itgam, Adgre1* (F4/80)*, Arg1, Mrc1, Cd14, Csf1r, Vegfa, Vcan, Cxcl2, Inhba, Pdcd1lg2* (PD-L2)*, Trem2, Havcr2, Ccr2, Cd14, Fabp5, Cd274* (PD-L1), *Cxcl1, Il1b* |
| 13 | *Lyz2, Cd68, Itgam, Adgre1* (F4/80), *Ccr2, Mrc1, Il1b, Cd14, Sell, Ly6c2, Csf1r, Vcan, Cxcl9, Apoe, Ly6a, Ly6i, Ms4a4c, Cxcl2, Inhba, Trem2, Tgfb1, Cd274* (PD-L1) |
| 15 | *Lyz2, Cd68, Itgam, Mrc1, Cd14, Csf1r, Cxcl9, Gatm, Ly6a, Ms4a4c, Trem2, Tgfb1, Adgre1* (F4/80), *Ccl8* |
| 17 | *Lyz2, Cd68, Itgam, Ccr2, Mrc1, Cd14, Csf1r, Gatm, Cxcl2, Inhba, Pdcd1lg2* (PD-L2)*, Trem2, Tgfb1, Ccl8, Lpl, Mgl2, Mki67, Lipa* |
| 18 | *Lyz2, Cd68, Itgam, Adgre1* (F4/80)*, Arg1, Mrc1, Nos2, Ccl8, Cd14, Csf1r, Apoe, Cxcl2, Pdcd1lg2* (PD-L2)*, Trem2, Folr2, Tgfb1, Cd274* (PD-L1), *Cxcl2, Fabp5, Lpl, Lipa* |

Table S3: Gene expression differences in myeloid, T cell and DC (dendritic cell) cell clusters. Expression changes (up-red/down-blue) are listed either in an individual cluster or in all clusters of either cell type (macrophage/monocytes, T cells, DC) combined. The latter include changes calculated based on average expression per combined clusters as indicated in Figure 2B.

Table S4: GO of gene expression changes listed in S Table 3. GO (gene ontology) categories for individual clusters and combined myeloid, T cell and DC (dendritic cell) clusters are shown.

Table S5: EC (endothelial cell) gene expression changes. Data for individual experiments (4 WT and 4 KO) are shown. One sheet (B) shows all gene expression data whereas the other (A) significant differences based on paired comparisons between WT and KO in individual experiments. Changes with either low WT or low KO are also presented in A as well as significantly different genes coding for ligands and receptors (C). WT= wild type, KO= *Shb* conditional deletion in EC.

Table S6: GO (gene ontology) categories of EC (endothelial cell) gene expresssion changes.

Table S7: Tumor and patient characteristics.

| patient | age | tumor classification | laterality | Size (mm) | status October 2024 | DFS years | OS years |
| --- | --- | --- | --- | --- | --- | --- | --- |
| 1 | 76 | T1bN0 | left | 7 | dead | 2 | 5 |
| 2 | 71 | T1N0 | left | 12 | dead | 2 | 3 |
| 3 | 68 | T1bN0 | left | 8 | alive | 4 | 4 |
| 4 | 73 | T2N0 | left | 43 | alive | 7 | 7 |
| 5 | 58 | T1cN1 micro  T1cN1 | left  right | 15  12 | dead | 1 | 3 |
| 6 | 84 | T2N1 | left | 24 | alive | 6 | 6 |
| 7 | 82 | T1N0 | left | 12 | alive | 3 | 3 |
| 8 | 40 | T2N1 | left | 35 | alive | 9 | 9 |
| 9 | 60 | T1N1 | right | 15 | alive | 10 | 10 |
| 10 | 60 | T1N1 | right | 20 | dead | 2 | 2 |
| 11 | 61 | T1N1 | right | 16 | alive | 10 | 10 |
| 12 | 82 | T2N1 | right | 22 | dead | 2 | 5 |
| 13 | 64 | T2N3 | right | 33 | alive | 3 | 6 |
| 14 | 62 | T2N1 | left | 36 | alive | 6 | 6 |
| 15 | 56 | T2N0 | left | 22 | alive | 5 | 5 |
| 16 | 60 | T2N1 | left | 21 | dead | 1 | 3 |
| 17 | 80 | T2N2 | right | 40 | dead | 2 | 8 |
| 18 | 84 | T2N1 | right | 30 | alive | 3 | 3 |
| 19 | 84 | T2N1 | right | 49 | alive | 1 | 3 |
| 20 | 37 | T2N1 | left | 23 | alive | 2 | 8 |

DFS: disease free survival, OS overall survival assesed in October 2024

Table S8: List of immune reagents and software.

Antibody target Host Supplier Catalog number Fluorophore

mouse CD31 rat BD bioscience 553370 none

mouse VE-cadherin goat R&D systems AF1002 none

mouse HIF1a rabbit Cell signaling 36169 none

Human VE-cadherin rabbit Cell signaling cs93467(E6N7A) none

Human FpA mouse Novus bio NB100-73043(49D2) none

Human CD4 mouse Dako M7310(4B12) none

Human FoxP3 rabbit Cell signaling 98377(D2W8E) none

Human CTLA4 rabbit Abcam ab251599(CAL49) none

Human CD163 mouse Novocostra NCL-L-CD163(10D6) none

Human CD8 rabbit Cell signaling 85336(D8A8Y) none

Human GrzB mouse Dako M7235(GrB-7) none

Human PD1 mouse Cell signaling 43248(EH33) none

Human PD-L1 rabbit Abcam ab251599(SP142) none

Human CD20 mouse Dako M0755(L26) none

Mouse CD31 rat Biolegend 102514 Alexa 488

Anti-mouse IgG horse Vectorlabs VEC-MP-7402 Immpress-HRP

Anti-rabbit IgG horse Vectorlabs VEC-MP-7401 Immpress-HRP

rat IgG donkey Invitrogen A21209 Alexa 594

goat IgG donkey Invitrogen A11055 Alexa 488

rabbit IgG donkey Invitrogen A21206 Alexa 488

Opal reagents NA Akoya FP1488001 570

Opal reagents NA Akoya FP1501001 780

Opal reagents NA Akoya FP1487001 520

Opal reagents NA Akoya FP1497001 690

Opal reagents NA Akoya FP1495001 620

Opal reagents NA Akoya FP1500001 480

Software

ImageJ: <https://github.com/imagej/imagej2>

Graphpad: <https://www.graphpad.com/>

Phenochart: <https://www.akoyabio.com/support/software/>

QuPath: <https://qupath.readthedocs.io/en/0.5/>

Cell Ranger: <https://www.10xgenomics.com/support/software/cell-ranger/latest>

Seurat package: <https://satijalab.org/seurat/>
