# Supplementary figures and images for: Reduced vascular leakage correlates with breast carcinoma T regulatory cell infiltration but not with metastatic propensity
Source: Mol Oncol. 2025 Oct 16;20(3):753–78. doi: 10.1002/1878-0261.70144 (PMC13042803; doi:10.1002/1878-0261.70144)

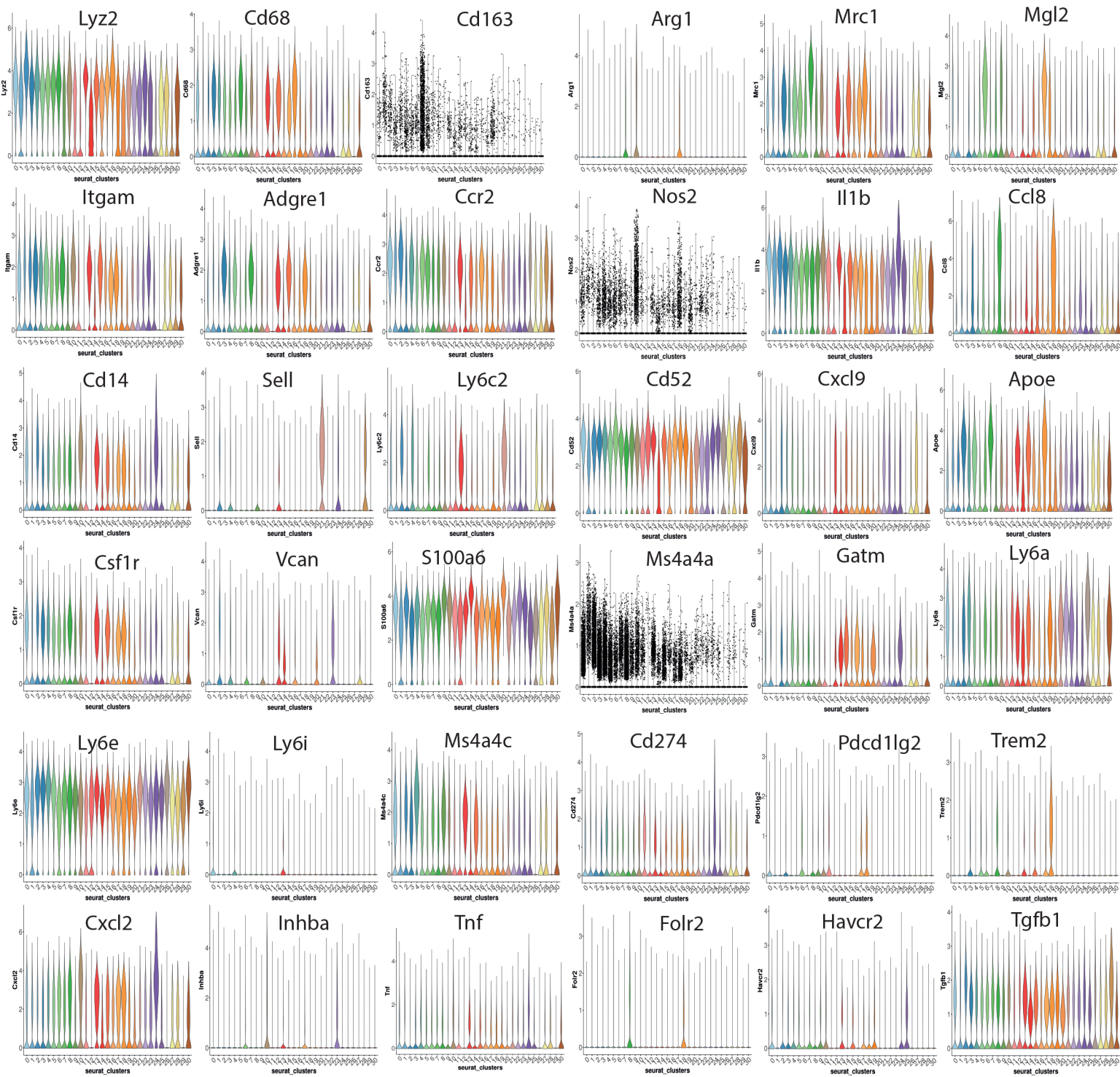

Supplement: Supplementary file 1 — Fig. S1. Cluster expression of signature macrophage and monocyte gene markers. Fig. S2. Cluster expression of signature T cell and DC (dendritic cell) gene markers. Fig. S3. (A) Typical staining for VE‐cadherin (vascular, red), FpA (leakage, gray), and CD4+‐cells (green). (B) DAPI of the same section. Tumor boundary has been indicated. (C) CD8 (white) and GZMB (green) or (D) PD1 (red). (E, F) CD163 (yellow) and PD‐L1 (red) in human breast cancer stroma. Fig. S4. Correlations between CD4 and FOXP3 (A), CTLA4 and CD4 (B) and CTLA4 and FOXP3/CD4 (C) in the human breast cancer cohort. Correlation between vascular density (VE‐cadherin staining) and CD4 (D), vascular density and FOXP3 (E) and vascular density and CTLA4 (F) is also shown. (G) Correlation CD4 and PD‐L1 and (H) correlation CD163 and PD‐L1. A tumor with tumor cell CTLA4‐staining is also shown. (I) DAPI (blue), (J) CD4 (green) and FOXP3 (red), (K) FpA (gray) and VE‐cadherin (red) and (L) CTLA4 (yellow). Table S1. IC cluster cell numbers. Table S2. Extended list of signature markers in different myeloid clusters. Table S3. Gene expression differences in myeloid, T cell and DC (dendritic cell) cell clusters. Table S4. GO of gene expression changes listed in Table S3. Table S5. EC (endothelial cell) gene expression changes. Table S6. GO (gene ontology) categories of EC (endothelial cell) gene expresssion changes. Table S7. Tumor and patient characteristics. Table S8. List of immune reagents and software. [file MOL2-20-753-s001.zip › mol270144-sup-0002-FigureS1.pdf]

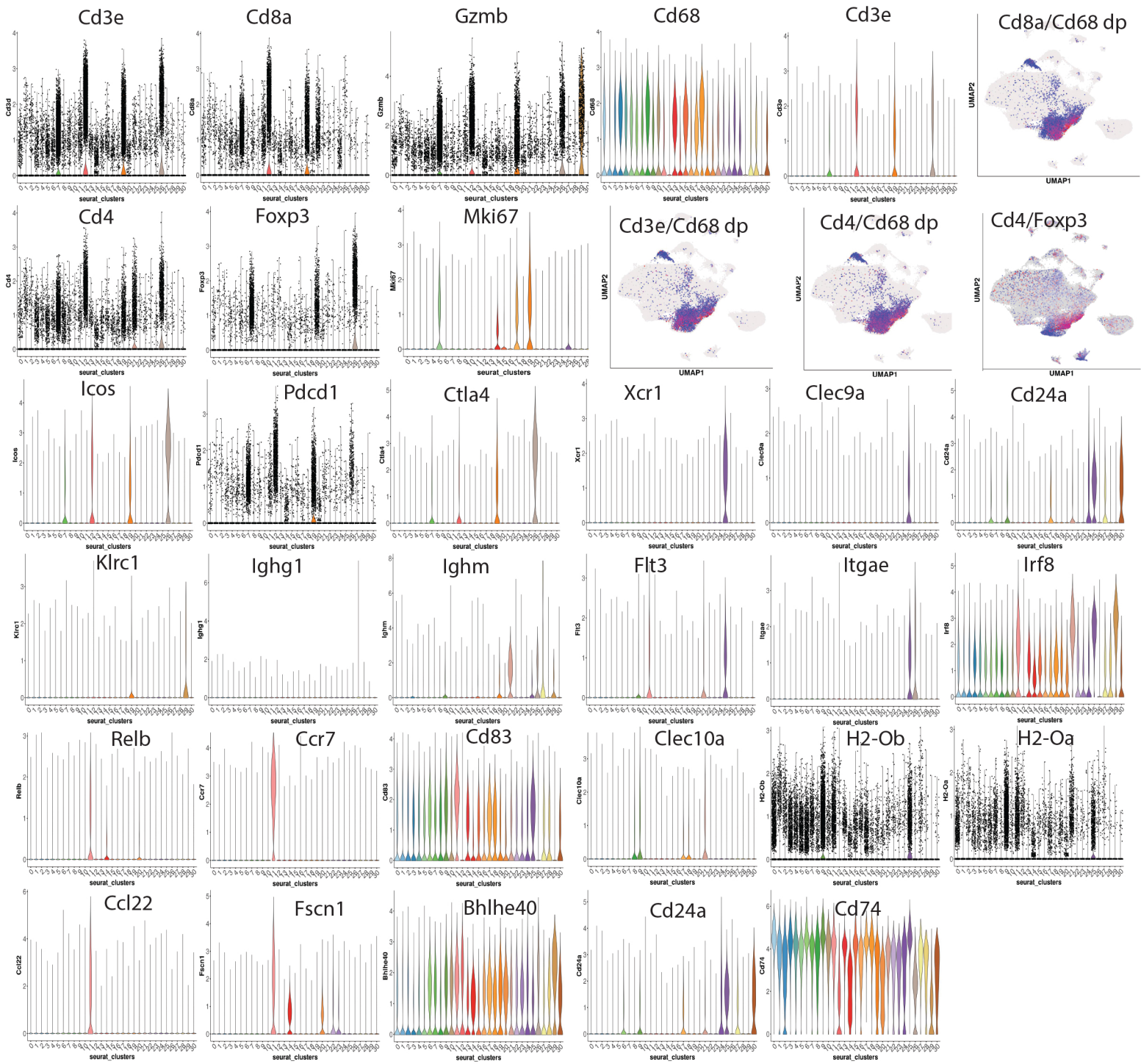

Supplement: Supplementary file 1 — Fig. S1. Cluster expression of signature macrophage and monocyte gene markers. Fig. S2. Cluster expression of signature T cell and DC (dendritic cell) gene markers. Fig. S3. (A) Typical staining for VE‐cadherin (vascular, red), FpA (leakage, gray), and CD4+‐cells (green). (B) DAPI of the same section. Tumor boundary has been indicated. (C) CD8 (white) and GZMB (green) or (D) PD1 (red). (E, F) CD163 (yellow) and PD‐L1 (red) in human breast cancer stroma. Fig. S4. Correlations between CD4 and FOXP3 (A), CTLA4 and CD4 (B) and CTLA4 and FOXP3/CD4 (C) in the human breast cancer cohort. Correlation between vascular density (VE‐cadherin staining) and CD4 (D), vascular density and FOXP3 (E) and vascular density and CTLA4 (F) is also shown. (G) Correlation CD4 and PD‐L1 and (H) correlation CD163 and PD‐L1. A tumor with tumor cell CTLA4‐staining is also shown. (I) DAPI (blue), (J) CD4 (green) and FOXP3 (red), (K) FpA (gray) and VE‐cadherin (red) and (L) CTLA4 (yellow). Table S1. IC cluster cell numbers. Table S2. Extended list of signature markers in different myeloid clusters. Table S3. Gene expression differences in myeloid, T cell and DC (dendritic cell) cell clusters. Table S4. GO of gene expression changes listed in Table S3. Table S5. EC (endothelial cell) gene expression changes. Table S6. GO (gene ontology) categories of EC (endothelial cell) gene expresssion changes. Table S7. Tumor and patient characteristics. Table S8. List of immune reagents and software. [file MOL2-20-753-s001.zip › mol270144-sup-0003-FigureS2.pdf]

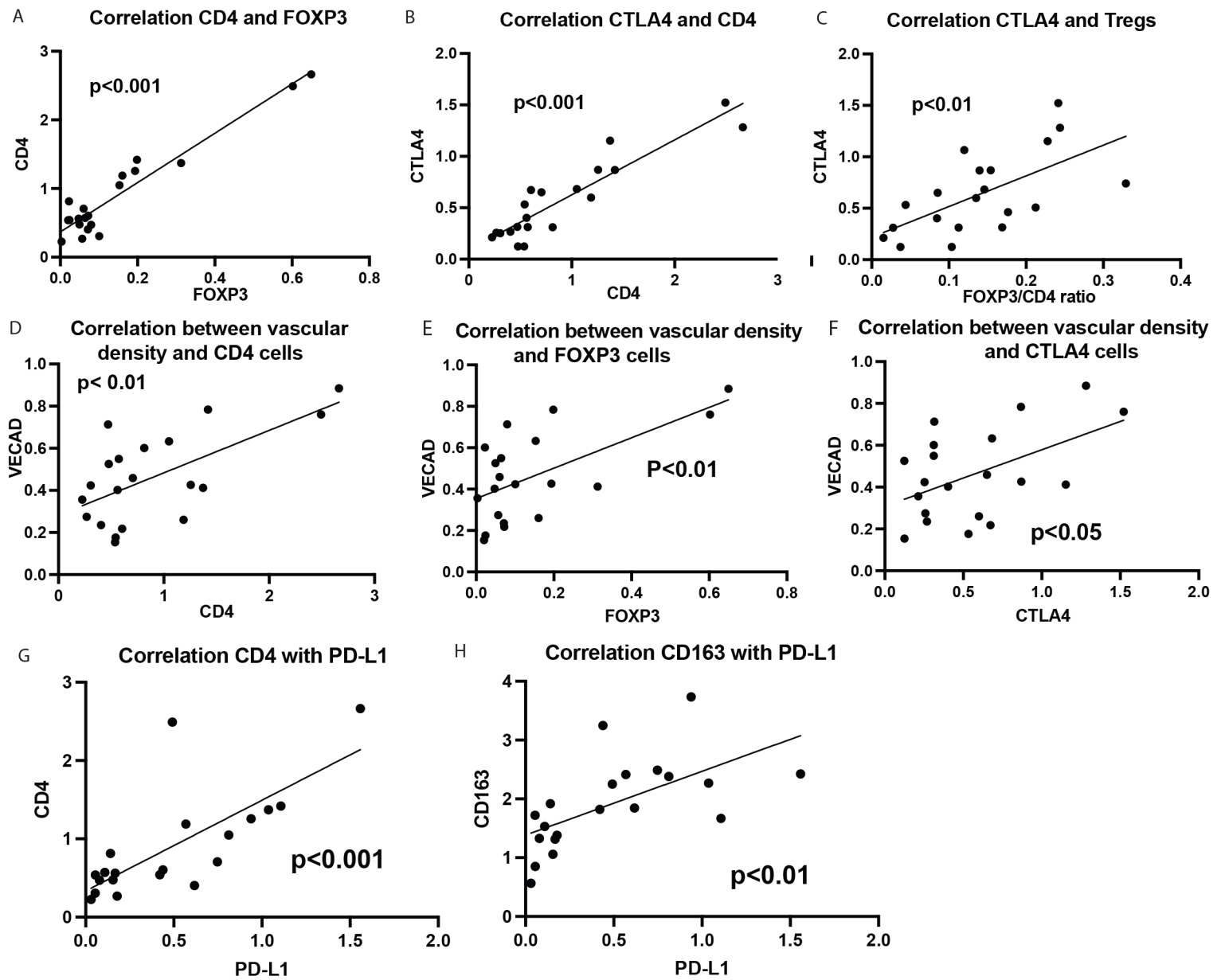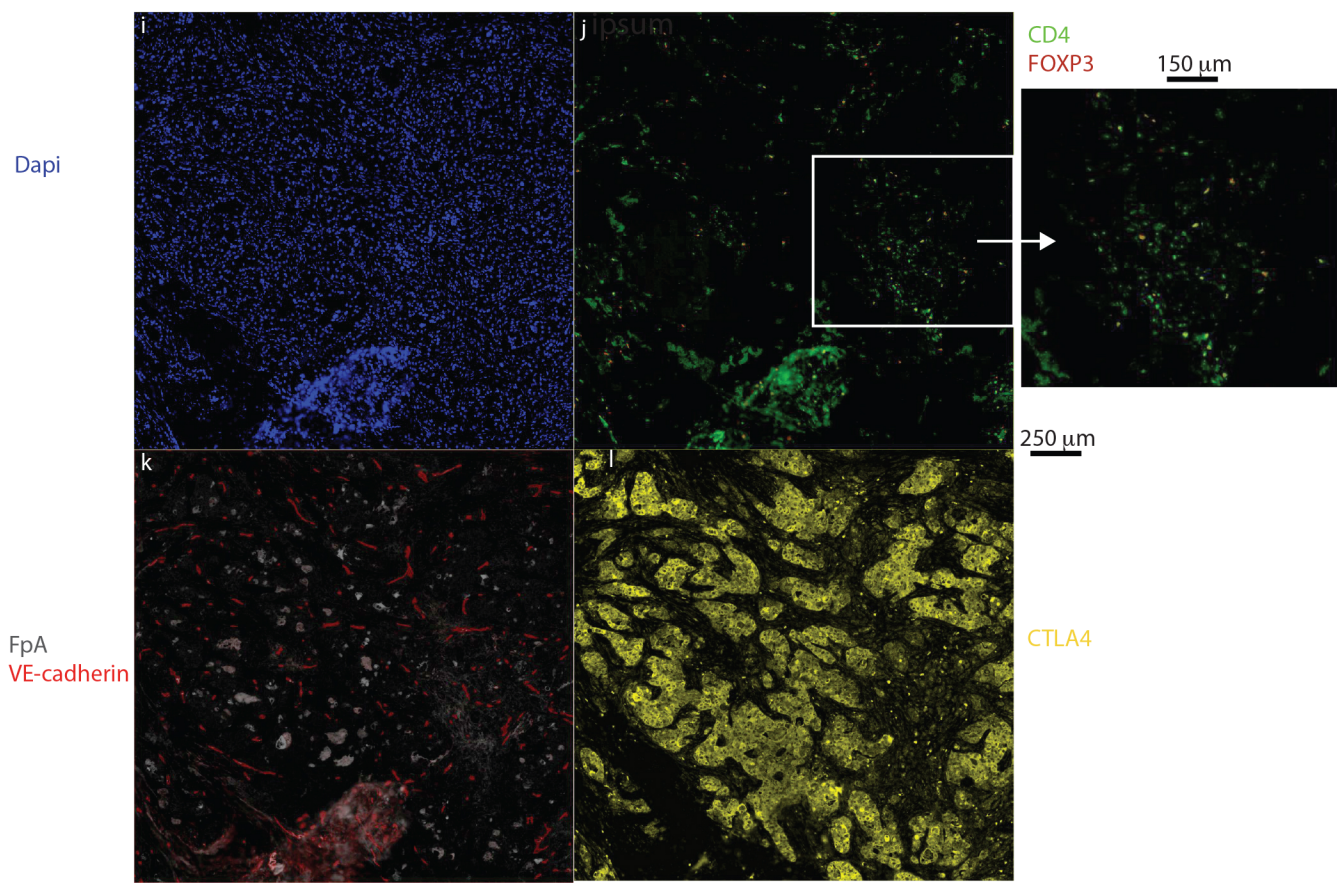

Supplement: Supplementary file 1 — Fig. S1. Cluster expression of signature macrophage and monocyte gene markers. Fig. S2. Cluster expression of signature T cell and DC (dendritic cell) gene markers. Fig. S3. (A) Typical staining for VE‐cadherin (vascular, red), FpA (leakage, gray), and CD4+‐cells (green). (B) DAPI of the same section. Tumor boundary has been indicated. (C) CD8 (white) and GZMB (green) or (D) PD1 (red). (E, F) CD163 (yellow) and PD‐L1 (red) in human breast cancer stroma. Fig. S4. Correlations between CD4 and FOXP3 (A), CTLA4 and CD4 (B) and CTLA4 and FOXP3/CD4 (C) in the human breast cancer cohort. Correlation between vascular density (VE‐cadherin staining) and CD4 (D), vascular density and FOXP3 (E) and vascular density and CTLA4 (F) is also shown. (G) Correlation CD4 and PD‐L1 and (H) correlation CD163 and PD‐L1. A tumor with tumor cell CTLA4‐staining is also shown. (I) DAPI (blue), (J) CD4 (green) and FOXP3 (red), (K) FpA (gray) and VE‐cadherin (red) and (L) CTLA4 (yellow). Table S1. IC cluster cell numbers. Table S2. Extended list of signature markers in different myeloid clusters. Table S3. Gene expression differences in myeloid, T cell and DC (dendritic cell) cell clusters. Table S4. GO of gene expression changes listed in Table S3. Table S5. EC (endothelial cell) gene expression changes. Table S6. GO (gene ontology) categories of EC (endothelial cell) gene expresssion changes. Table S7. Tumor and patient characteristics. Table S8. List of immune reagents and software. [file MOL2-20-753-s001.zip › mol270144-sup-0005-FigureS4.pdf]
